# Tunable Platform Capacity of Metal–Organic Frameworks via High-Entropy Strategy for Ultra-Fast Sodium Storage

**DOI:** 10.1007/s40820-025-01706-3

**Published:** 2025-03-26

**Authors:** Shusheng Tao, Ziwei Cao, Xuhuan Xiao, Zirui Song, Dengyi Xiong, Ye Tian, Wentao Deng, Youcai Liu, Hongshuai Hou, Guoqiang Zou, Xiaobo Ji

**Affiliations:** 1 Present Address: College of Chemistry and Chemical Engineering, Central South University, Changsha, 410083 People’s Republic of China; 2 Department of Chemistry, University College London, London, WC1H 0AJ UK; 3 Department of Materials, University of Oxford, Oxford, OX1 3PH UK

**Keywords:** High-entropy metal-organic frameworks, Fast-charging materials, Sodium-ion batteries, Sodium-ion capacitors

## Abstract

Precise regulation of the platform capacity/voltage of electrode materials contributes to the efficient operation of sodium-ion fast-charging devices. However, the design of such electrode materials is still in a blank stage. Herein, based on tunable metal–organic frameworks, we have designed a novel material system—two-dimensional high-entropy metal–organic frameworks (HE-MOFs), which exhibits unique properties in sodium storage and is of vital importance for realizing fast-charging batteries. Furthermore, we have found that the high-entropy effect can regulate the electronic structure, the sodium-ion migration environment, and the sodium-ion storage active sites, thereby meeting the requirements of electrode materials for sodium-ion fast-charging devices. Impressively, the HE-MOFs material still maintains a reversible specific capacity of 89 mAh g^−1^ at a current density of 20 A g^−1^. It presents an ideal sodium storage voltage plateau of approximately 0.5 V, and its platform capacity is increased to 122.7 mAh g^−1^, far superior to that of Mn-MOFs (with no platform capacity). This helps to reduce safety hazards during the fast-charging process and demonstrates its great application value in the fields of fast-charging sodium-ion batteries and capacitors. Our research findings have broken the barriers to the application of non-conductive MOFs as energy storage materials, enhanced the understanding of the regulation of platform capacity and voltage, and paved the way for the realization of high-security sodium-ion fast-charging devices.

**Supplementary Information:**

The online version contains supplementary material available at 10.1007/s40820-025-01706-3.

## Highlights

- A novel high-entropy metal–organic frameworks (HE-MOFs) electrode for fast sodium-ion storage devices has been realized by introducing five metallic elements.
- The platform capacity/voltage of the electrode materials are precisely regulated by the adjustable metal species/content of HE-MOFs.
- The sodium-ion capacitors assembled based on high-entropy MOFs electrode exhibit high-power density (20,000 W kg^-1^) and high-energy density (99.4 Wh kg^-1^).

**Supplementary Information:**

The online version contains supplementary material available at 10.1007/s40820-025-01706-3.

## Graphical abstract

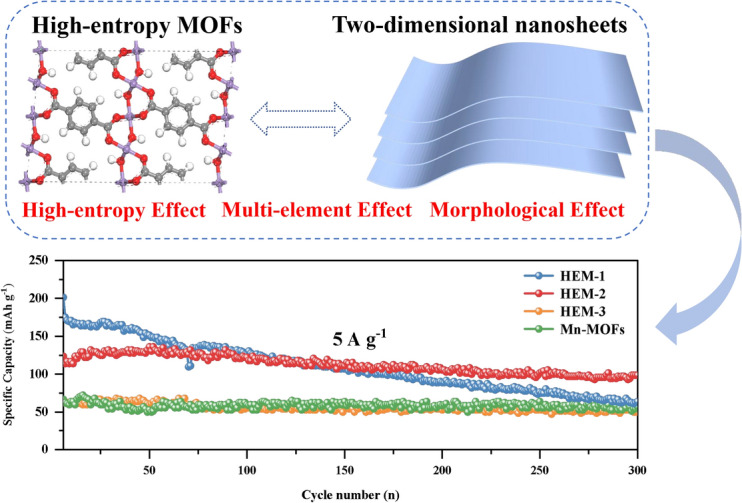

## Introduction

The development and application of advanced materials have provided a constant impetus to improve the energy/power density of energy storage devices [1–4]. In recent decades, the widespread use of lithium-ion batteries has propelled the development of human civilization [5–7]. However, the limited availability of lithium resources constrains the widespread use of lithium-ion batteries in large-scale fast energy storage devices [8, 9]. Sodium-ion energy storage devices, characterized by their low cost, abundant raw materials, and more evenly distributed global sodium resources, represent a better solution for large-scale fast energy storage [10, 11]. Nevertheless, developing sodium-ion energy storage devices with both rapid charging capability and high-energy density poses significant challenges. During fast charging, sodium ions may precipitate in metallic form, thereby affecting the safety performance of the entire battery [12, 13]. Meanwhile, the electrode material, which is the "heart" of the energy storage device, exhibits an uncontrollable voltage plateau and platform capacity. The realization of controllable voltage plateau and plateau capacity is conducive to the reduction of the risk factor in the operation of sodium-ion energy storage devices and the improvement of their energy/power density.

Metal–organic frameworks with adjustable metal components and variable contents provide the possibility of regulating the voltage platform and platform capacity [14–16]. In recent years, the development of conductive metal–organic frameworks (MOFs) has expanded the prospects of direct application of MOFs in energy storage [17–19]. However, the synthesis of conductive MOFs is complex, costly, and limited in variety [20–22]. On the contrary, non-conductive MOFs are numerous and simple to synthesize, and their derivatives are widely used in energy storage [23, 24]. Nevertheless, their poor chemical stability and conductivity seriously hinder their direct use as electrode materials [25]. In disordered multi-component systems, a large conformational entropy can often stabilize the crystal structure and promote its chemical and structural diversity [26–30]. To date, a large number of high-entropy materials, such as initial high-entropy alloys (HEAs) and subsequently high-entropy oxides (HEOs), have been used in applications such as environmental protection, electrochemical energy storage, and catalysis [31–35]. Whether high-entropy (HE) strategies can effectively modify non-conductive MOFs materials and induce them to become excellent sodium fast storage electrode materials deserves in-depth investigation. Back in 2014, Omar M. Yaghi et al. [36] had already synthesized a series of MM-MOF-74 materials (Mg, Ca, Sr, Ba, Mn, Fe, Co, Ni, Zn, and Cd) containing 2/4/6/8/10 metal ions by hydrothermal methods. All metal ions are present in the MOF-74 lattice, and no heterogeneous phases are produced. Additionally, in the past 3 years, a small number of high-entropy Prussian blue materials have been synthesized and investigated for their electrochemical performance advantages in energy storage systems, such as sodium-ion batteries and lithium–sulfur batteries [11, 32, 37]. Although a small number of high-entropy MOFs have been synthesized, their reported occurrences are in the single digits, which is due to the fact that the synthesis mechanism of high-entropy MOFs is still unclear, and the synthesis of high-entropy MOFs is still a major challenge. Furthermore, the charge compensation mechanism of high-entropy MOFs applied to sodium-ion rapid storage electrode materials remains unclear, and whether elemental effects can effectively regulate the electrode material's plateau voltage and plateau capacity is also uncertain, warranting further investigation.

In this work, we introduced four additional metal elements, Co, Ni, Cu, and Zn, into the Mn-MOFs, successfully synthesizing two-dimensional high-entropy MOFs. Through density functional theory (DFT) simulations, time-of-flight secondary ion mass spectrometry, synchrotron radiation, and other characterization methods, we investigated charge compensation mechanisms of high-entropy MOFs as fast sodium storage anodes. Meanwhile, the respective energy storage advantages of different elements (Co, Ni, Cu, and Zn) in Mn-MOFs were revealed. By adjusting the ratios of different metal elements, precise regulation of the platform voltage and capacity is achieved. The synergistic effect of the five elements in the high-entropy MOFs allows the designed high-entropy MOFs to exhibit unique rate performance advantages. The designed high-entropy MOFs material delivers a reversible specific capacity of 89 mAh g^−1^ at an ultra-high current density of 20 A g^−1^ and exhibits an ideal plateau voltage, reducing the risk of sodium-ion precipitation in metallic form. Both sodium-ion batteries and sodium-ion capacitors assembled with high-entropy MOFs materials as anode provide excellent rate performance and long cycle stability. We introduced the concept of high-entropy into non-conductive MOFs and successfully designed two-dimensional high-entropy MOFs. This provides theoretical guidance for non-conductive MOFs to be directly utilized as rapid and safe sodium storage electrode materials, breaking down barriers for their direct use as electrode materials.

## Results and Discussion

### Theoretical Calculations

**Fig. 1:**
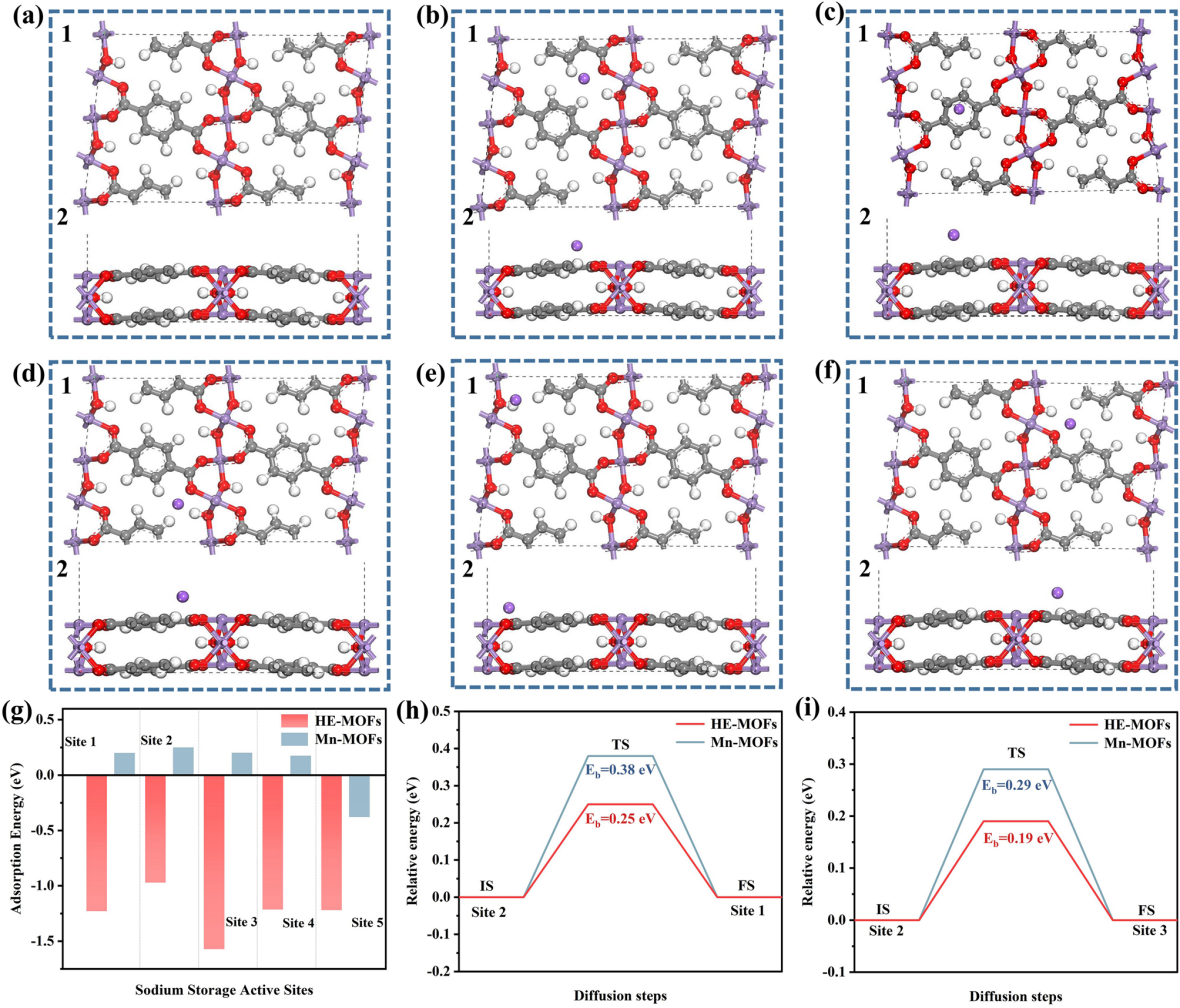
Theoretical simulation calculations of high-entropy MOFs (Elemental Mn at 60 percent) and Mn-MOFs: **a1** Top and **a2** main views of schematic crystal structure of high-entropy MOFs. Sodium storage active sites of high-entropy MOFs: **b1** Top and **b2** main views of site 1. **c1** Top and **c2** main views of site 2. **d1** Top and **d2** main views of site 3. **e1** Top and **e2** main views of site 4. **f1** Top and **f2** main views of site 5. **g** Adsorption energy of sodium ions at different sites in MOFs materials. **h**, **i** Migration energy barriers of high-entropy MOFs and Mn-MOFs

In order to theoretically investigate in depth the effect of different metal element contents on the formation of high-entropy MOFs and the kinetics of sodium storage, DFT calculations were used. Firstly, the binding energies of Mn sites in Mn-MOFs substituted by different metal elements were calculated, and it was demonstrated that elements such as Co, Ni, Cu, and Zn could theoretically occupy the Mn sites, resulting in the formation of high-entropy MOFs containing five metal elements (Fig. S1). Crystal structures of Mn-MOFs as well as high-entropy MOFs were constructed, while the variation of electron density in the crystal structures was further analyzed (Figs. 1a and S2-S4). The crystal structures of high-entropy MOFs were constructed with Mn content at 80%, 60%, and 40%, respectively. The other four metal elements (Co, Ni, Cu, and Zn) are presented in a ratio of 1:1:1:1. The electron cloud density of MOFs changes as the manganese content decreases and the content of the other four metal elements increases, demonstrating that an increase in the content of the four metal elements favors the electrical conductivity of the material (Figs. S2-S4). In order to accurately understand the difference in conductivity between high-entropy MOFs and Mn-MOFs materials, we carried out density of states and energy band gap analyses for Mn-MOFs, high-entropy MOFs with 60% Mn content. As shown in Fig. S5, the Mn-MOFs exhibits semiconducting properties, and the high-entropy MOFs do not display obvious energy band gaps and exhibit metal-like properties, which demonstrates that the chaotic entropy effect can improve the electrical conductivity of the materials [38]. In order to further investigate the energy storage mechanism of high-entropy MOFs, the sodium storage active sites were analyzed by theoretical calculations, taking high-entropy MOFs (Mn content of 60%) as an example. As exhibited in Fig. 1b-f, the energy storage sites of high-entropy MOFs are mainly dominated by organic ligands, which are classified into five sites. The adsorption energies of Mn-MOFs and high-entropy MOFs at the above five active sites were calculated separately. As shown in Fig. 1g, the adsorption energies of Mn-MOFs at the first four active sites were positive, demonstrating that the adsorption process of sodium ions at these active sites could not proceed spontaneously. Compared with Mn-MOFs, the adsorption energies of high-entropy MOFs at the active sites are all negative, demonstrating that the introduction of multiple metal elements lowers the sodium-ion adsorption energy barriers, which favors the adsorption and storage of sodium ions. In order to further verify that the high-entropy MOFs offer excellent rate performance, the migration energy barriers of sodium ions from near site 2 to near site 1 and from near site 2 to near site 3 were calculated (Fig. S6). Calculations indicate that high-entropy MOFs deliver a smaller migration energy barrier compared to Mn-MOFs, which facilitates the migration of sodium ions in the bulk phase (Fig. 1h, i). Theoretically, high-entropy MOFs exhibit excellent electronic conductivity, low sodium-ion adsorption energy, and small sodium-ion migration energy barriers, which will improve the sodium rate storage performance of MOFs materials.

### Material Characterization

**Fig. 2:**
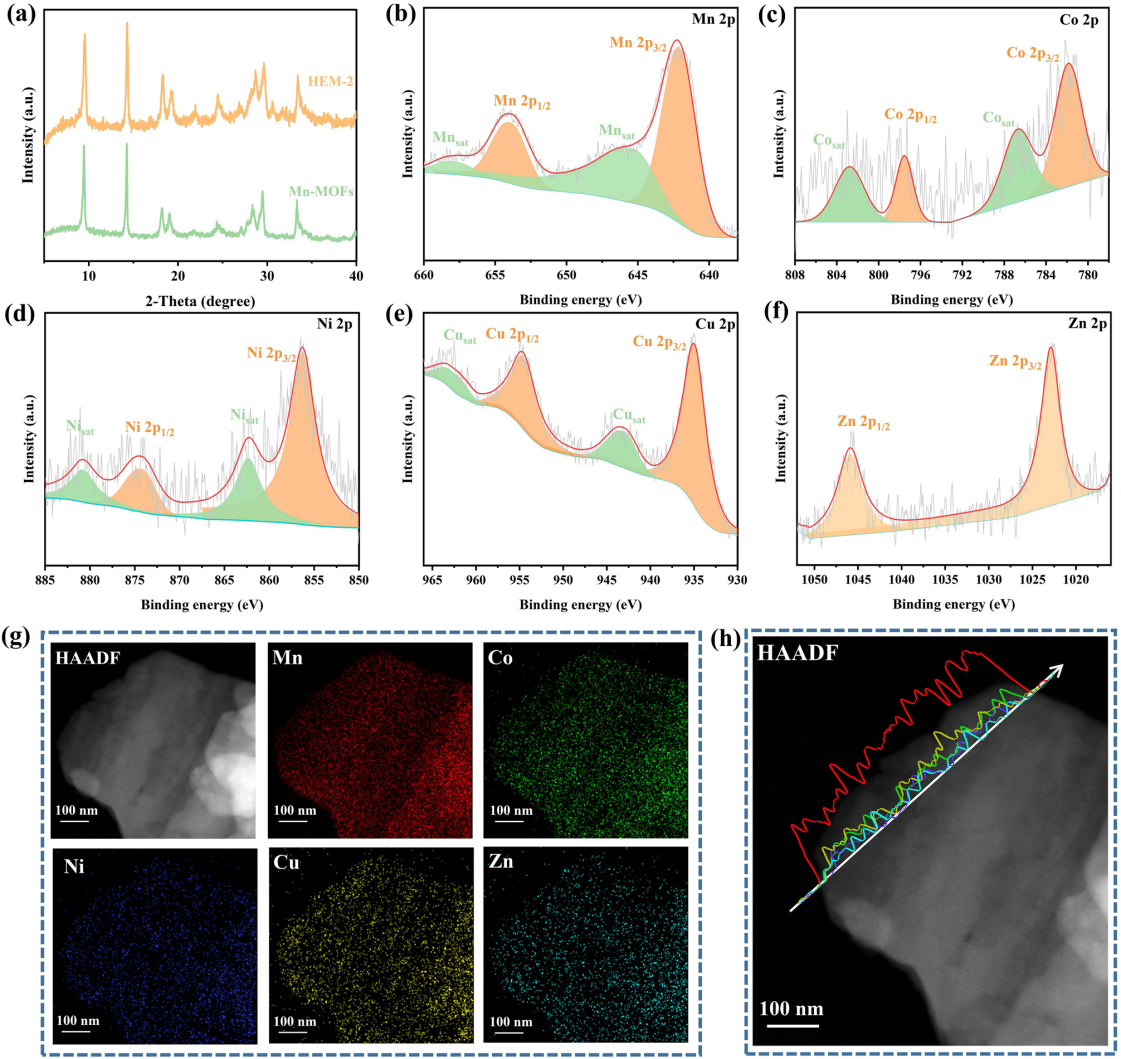
Microscopic morphological characterization: **a** XRD and **b-f** XPS spectrum of Mn 2*p*, Co 2*p*, Ni 2*p*, Cu 2*p*, and Zn 2*p* of HEM-2, respectively. **g** Elemental mappings (Mn, Co, Ni, Cu, and Zn) of HEM-2. **h** Elemental (Mn, Co, Ni, Cu, and Zn) line scanning of HEM-2 (Inside: elemental content variation curves, where the line colors correspond to the elements in the elemental mappings)

Inspired by the synthesis of Mn-MOFs and theoretical calculations, high-entropy MOFs are fabricated by mixed five kinds of other metal central ions with different ratios, which is named HEM-1 with 40% manganese content, HEM-2 with 60% manganese content, and HEM-3 with 80% manganese content. As illustrated in Table S1, the inductively coupled plasma atomic emission spectrometry (ICP-AES) analysis reveals that the atomic ratios of Mn/Co/Ni/Cu/Zn in HEM-1/2/3 are 4.3/1.3/1.9/1.7/0.8, 6.2/0.7/1.0/1.4/0.7, and 8.4/0.3/0.6/0.6/0.1, respectively, in which the percentage of Mn is approximately consist with the feed ratio, while the content of Ni and Cu is always slightly higher than the feed ratio, and the content of Co and Zn is always slightly lower. The above phenomenon may be caused by the different coordination abilities of different central metal ions in the coordination process. To further demonstrate the successful synthesis of high-entropy MOFs, the synthesized MOFs materials were tested by X-ray diffraction. The characteristic peaks of the prepared samples were consistent with those of Mn-MOFs with no spurious peaks, demonstrating that its structure conformation is in agreement with the Mn-MOFs (Figs. 2a and S7) [39]. In the Raman spectrum, Mn-MOFs and HEM-1/2/3 exhibit five strong bands, which are consistent with the vibrational fingerprint of the benzene ring. The peaks at 1610, 1420, and 1135 cm^−1^ correspond to the in-plane vibrational mode of the benzene ring. It is also clear that the two peaks at 859 and 632 cm^−1^ are related to the out-of-plane deformation of C-H (Fig. S8a, b). The FTIR spectra of Mn-MOFs and HEM-1/2/3 present similar peaks as expected indicate that the organic fraction of these four samples is basically the same (Fig. S8c, d). The broad and strong peak near 3440 cm^−1^ is an extension of hydroxyl or water molecules. The peaks at 1551 and 1400 cm^−1^ correspond to C = C stretching vibrations of mononuclear aromatics, representing skeletal vibrations of benzene rings. The signal at 1103 cm^−1^ indicates C-O single bond stretching vibrations, while the range of 700–900 cm^−1^ exhibits C-H in-plane and out-plane swinging vibration peaks associated with benzene rings. Additionally, the peak at 543 cm^−1^ is attributed to vibrations related to the Mn–O bond or TM-O (TM = Co, Ni, Cu, and Zn).

X-ray photoelectron spectroscopy (XPS) can be used to analyze elemental species and their valence states on the surface of high-entropy MOFs materials. Consequently, XPS characterization and analysis were conducted on HEM-2 materials. The XPS spectrum of Mn 2*p* showed two pairs of characteristic peaks within the binding energy range of 638–660 eV (Fig. 2b). Among them, the characteristic peaks located at 642.2 as well as 654.05 eV were attributed to Mn 2*p*_3/2_ and Mn 2*p*_1/2_, respectively, and the other pair of broad peaks corresponded to the satellite peaks of the Mn element. The XPS spectrum of Co 2*p* could be fitted to four characteristic peaks (Fig. 2c). Two characteristic peaks at about 781.8 and 797.6 eV can be attributed to Co 2*p*_3/2_ and Co 2*p*_1/2_, while two broad peaks at about 786.7 and 802.9 eV can be correlated with the satellite peaks of Co element. The binding energies of Ni 2*p*_3/2_ and Ni 2*p*_1/2_ are 856.5 and 874.6 eV, suggesting that the Ni^2+^ in the Ni–O bond (Fig. 2d). As shown in Fig. 2e, the main peaks of Cu 2*p* are located at 935.1 eV (Cu 2*p*_3/2_) and 954.9 eV (Cu 2*p*_1/2_), corresponding to the peak of divalent copper. The two main peaks of element Zn can be ascribed to divalent zinc (Fig. 2f). As shown in Fig. S9, soft X-ray synchrotron radiation characterization reveals the valence states of elements Mn, Co, Ni, and Cu, offering additional confirmation of the synthesis of high-entropy MOFs. Meanwhile, desktop synchrotron radiation technology and hard X-ray synchrotron radiation technology were employed to demonstrate the presence of elements such as Mn, Co, Ni, Cu, and Zn in the designed high-entropy MOFs. As shown in Fig. S10a-d, distinct absorption peaks are observed for the K-edges of Mn, Co, Ni, and Zn. As illustrated in Fig. S10e-i, the hard X-ray absorption spectra further confirm the existence of Mn, Co, Ni, Cu, and Zn, with each element displaying absorption edges distinct from those of their respective metals. A series of characterization techniques validate the synthesis of high-entropy MOFs.

Figure S11a-h shows scanning electron microscope (SEM) images of Mn-MOFs and HEM-1/2/3, which both can be observed the layered stacked structure. Transmission electron microscopy (TEM) images of HEM-2 and Mn-MOFs in Fig. S11i, j illustrated layered structure consistent with SEM. As displayed in Figs. S11k and 2g, elemental mappings exhibit the distribution of Mn, C, O elements and Mn, Co, Ni, Cu, Zn. The elements of Mn, C, and O are uniformly distributed on the surface Mn-MOFs, and the elements of Mn, Co, Ni, Cu, and Zn are uniformly distributed in HEM-2, which proves the successful replacement of the central metal ion. Linear scans of the elements (Mn, Co, Ni, Cu, and Zn) in the HEM-2 (Fig. 2h) indicate that there are distinct signal peaks for all five metal elements, and they are in accordance with the designed metal ratios. Then, the thickness of the nanosheets has been measured by atomic force microscope (AFM), which is ultra-thin with approximately 1 nm. This result indicates that the substitution of other metal centers will not affect the thickness of the nanosheets (Fig. S12). Two-dimensional morphology can effectively shorten the distance for sodium-ion charge transfer, promote sodium-ion transport, and facilitate the enhancement of surface pseudo-capacitance. To further investigate the function of each metal center in the charging and discharging process, four bimetallic MOFs, namely, MnCo-MOFs, MnNi-MOFs, MnCu-MOFs, and MnZn-MOFs, were designed. The ICP-AES reveals that atomic ratios of Mn to M (M = Co/Ni/Cu/Zn) in them are about 6.68/3.32, 6.61/3.39, 5.20/4.80, and 7.65/2.35, respectively (Table S2). Simultaneously, the bimetallic MOFs prepared for comparison also display the same characteristic peaks (Fig. S13a). Meanwhile, the Raman characteristic peaks of bimetallic MOFs are consistent with those of Mn-MOFs, demonstrating the successful synthesis of bimetallic MOFs (Fig. S13b).

### Electrochemical Performance

**Fig. 3:**
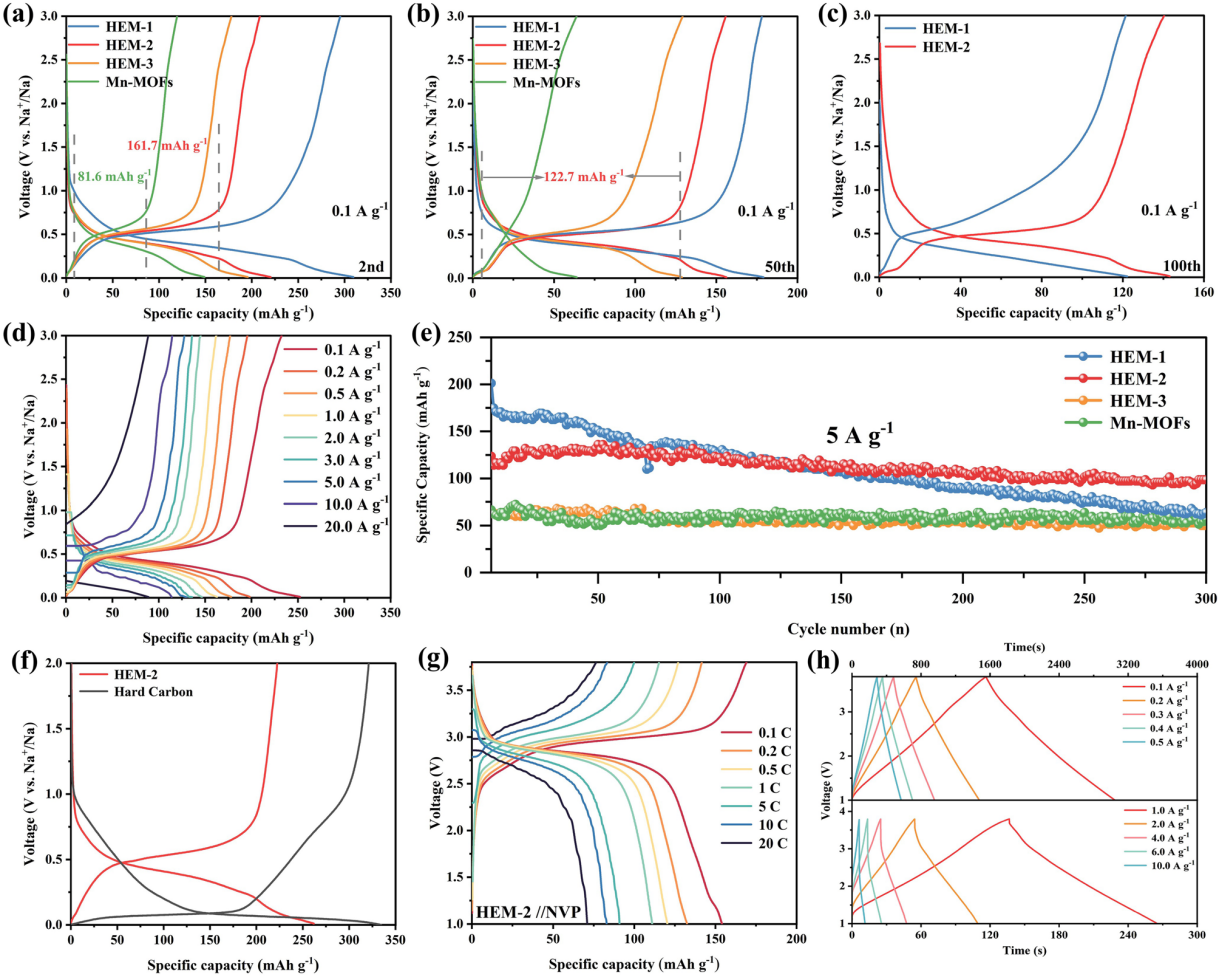
Electrochemical performance. **a** comparison of the 2nd lap charge/discharge curves and **b** comparison of the 50th lap charge/discharge curves of HEM-1/2/3 and Mn-MOFs. **c** Comparison of the 100th lap charge/discharge curves of HEM-1/2. **d** Discharge–charge profiles of HEM-2 at different current densities. **e** Long cycle performance of MOFs materials. **f** Discharge–charge profiles of HEM-2 compared with hard carbon materials at 0.1 A g^−1^. **g** Discharge–charge profiles of full sodium-ion batteries (HEM-2//NVP) at different current densities (1 C = 100 mA g^−1^). **h** GCD data for sodium-ion capacitors at different current densities (HEM-2//NHPAC with mass ratio of 1:2)

To further verify the electrochemical energy storage advantages of the high-entropy effect, the synthesized MOFs materials were evaluated as sodium anode materials in CR2016 coin-type cells. To investigate the redox reactions of sodium storage, cyclic voltammetry (CV) curves of high-entropy MOFs were measured at scan rates of 0.1 mV s^−1^ between 0.01 and 3.0 V (Fig. S14a-c). It is worth noting that the CV curves for the three high-entropy MOFs materials are similar, all showing two pairs of redox peaks below 0.5 V. In the first cathodic scan, the peak potential of the reduction peak is lower than the peak potential of subsequent scans, which may be due to the generation of solid–electrolyte interphase (SEI). All peaks remain unchanged after the first cycle, confirming the existence of a reversible and stable electrochemical reaction [40]. Figure S14e-g displays the constant current discharge–charge curve of the cell at 0.1 A g^−1^. All three high-entropy MOFs display voltage plateaus below 1 V and exhibit poor first coulomb efficiency, which is related to the generation of SEI films [41]. Notably, the first Coulombic efficiency of the high-entropy MOFs increases as the elemental manganese content decreases. As shown in Fig. S15a, the reversible specific capacities of the high-entropy MOFs are all higher than those of the Mn-MOFs at a current density of 0.1 A g^−1^. The reversible specific capacity of the high-entropy MOFs increases with increasing levels of Co, Ni, Cu, and Zn. However, the higher content of the four elements leads to a lower content of Mn, which may induce structural instability of the MOFs and thus further lead to poor cyclic stability. In order to further visualize the intrinsic relationship between the plateau voltage/capacity and the metal element content, the 2nd and 50th charge/discharge curves of HEM-1/2/3 and Mn-MOFs were analyzed. As shown in Fig. 3a, both high-entropy MOFs and Mn-MOFs exhibit obvious charging and discharging platforms. With the reduction of Mn element, the platform capacity increases, in which the charging platform capacity of HEM-2 is almost twice as much as that of Mn-MOFs, which suggests that the introduction of different metal elements (Co, Ni, Cu, and Zn) is beneficial to improve the platform capacity of MOFs materials. In addition, the platform voltage difference between the charging and discharging curves of the three high-entropy MOFs gradually decreases as the content of Mn element decreases, which demonstrates that the increase in the content of the other four metal elements is beneficial to reduce the polarization and improve the stability of the batteries. After 50 electrochemical cycles, the high-entropy MOFs still display an obvious charging and discharging plateau, among which, the charging plateau capacity of HEM-2 is 122.7 mAh g^−1^, while the charging and discharging plateau of the Mn-MOFs disappeared, which further demonstrates that the introduction of other metal elements has a favorable effect on the reversibility of the reaction (Fig. 3b). It is noteworthy that the plateau capacity of the HEM-1 material decays significantly and its polarization voltage increases. Further comparison of the HEM-1/2 charge/discharge curves after 100 electrochemical cycles reveals that the HEM-2 material exhibits a larger plateau capacity and smaller polarization voltage, which demonstrates that too low a manganese content is also detrimental to the electrochemical stability of the MOFs material (Fig. 3c). Figure S15b displays the rate performances of high-entropy MOFs and Mn-MOFs materials at various current densities from 0.1 to 20 A g^−1^. Compared with Mn-MOFs, HEM-2 exhibits excellent rate performance, which is related to the synergistic effect of multiple metal elements. Specifically, different metal elements may lead to different energy storage properties, and in addition, the presence of multiple elements alters the electronic structure, sodium-ion migration environment, and adsorption capacity of the metal–organic frameworks to improve the sodium-ion migration capacity in the materials. The charge–discharge curves of HEM-2 materials are shown in Fig. 3d. The nearly straight charge/discharge curves at an ultra-high current density of 20 A g^−1^ demonstrate that the HEM-2 material exhibits pseudo-capacitive behavior (reversible specific capacity of 89 mAh g^−1^), which may also be related to the two-dimensional morphology of the HEM-2 material. The same phenomenon occurs in high current density long cycle tests as in low current tests, and the HEM-2 also exhibits excellent stability (84.6% capacity retention after 300 cycles at a current density of 5 A g^−1^). Therefore, it can be concluded that the high-entropy MOFs material with Mn content of 60% exhibits large plateau capacity, small polarization voltage, excellent rate properties, and cycling stability. Meanwhile, the charging and discharging platform of HEM-2 material is significantly higher than that of hard carbon materials (Fig. 3f), and the increased voltage platform reduces the precipitation of sodium metal, which reduces the safety hazards such as short circuit caused by sodium dendrites, and is conducive to the improvement of the safety performance of fast-charging devices.

Although the transformation of the migration energy barrier revealed by DFT is instructive in elucidating the structural origin of the superior rate properties of high-entropy MOFs. However, these analyses only determine the bulk properties of the material and do not fully reflect the effect of the specific surface area of porous two-dimensional MOFs materials on the electrochemical properties. Moreover, in order to further verify that the high-entropy MOFs materials have fast charging and discharging performance, pseudo-capacitance test analysis was carried out. The mechanism of electrochemical reactions (i.e., charge transfer at the anode) is divided into Faraday and non-Faraday processes [42, 43]. Faraday processes store charge through redox reactions that occur within the active electrode material, which constrains fast charging due to the need for sodium ions to migrate into the bulk phase [44]. In contrast, non-Faraday processes store charge at the surface without carrier ion diffusion. Therefore, two-dimensional MOFs with porous nanostructures facilitate fast charging when non-Faraday reactions occur at electrodes. In order to quantify the pseudo-capacitive contribution of the two-dimensional MOFs material, CV tests were performed at different scan rates (0.1–1.0 mV s^−1^) in the voltage interval from 0.01 to 3.0 V (Fig. S16a, b). The CV curves of HEM-2 are in agreement with those of the Mn-MOFs. The pseudo-capacitive contribution at a fixed voltage can be calculated by the equation: $i(V) = k_{1} v + k_{2} v^{1/2}$[45]. The capacity of *k*_1_*v* corresponds to the non-Faraday process, and the capacity of *k*_2_*v*^1/2^ corresponds to the Faraday process. For instance, the non-Faraday capacity occupation of HEM-2 at 1.0 mV s^-1^ is presented in Fig. S16c. The pseudo-capacitive contributions of Mn-MOFs and HEM-2 are calculated in turn, and the contribution percentages are shown in Fig. S16d. The pseudo-capacitance contribution of HEM-2 is significantly higher than that of Mn-MOF, and the pseudo-capacitance percentage can reach up to more than 80%, which again provides theoretical support for the superior rate performance of high-entropy materials. In conclusion, the outstanding rate performance of HEM-2 may result from 1) the short diffusion distance of the two-dimensional MOFs nanostructure, 2) low migration energy barriers induced by entropic effects, and 3) the high pseudo-capacitive ratio induced by the surface area of the two-dimensional morphology [46, 47].

### Sodium Full Ion Batteries and Capacitors

To further validate the practical application potential of high-entropy MOFs materials, sodium-ion full cells were assembled with Na_3_V_2_(PO_4_)_3_ as the cathode material, and sodium-ion capacitors were assembled with activated carbon as the cathode material. Specific synthesis protocols for the Na_3_V_2_(PO_4_)_3_ materials and activated carbon materials (NHPAC) are reported in our previous article [48, 49]. The electrochemical properties and data analysis of the two cathode materials are elaborated in the Supplementary Information (Fig. S17). A sodium-ion full cell was designed with a cathode-to-anode mass ratio of 3:1, determined based on the reversible capacity of the anode. As shown in Fig. S18a, the assembled sodium-ion full cell presents a pair of strong redox peaks due to the clearly visible electrochemical reaction platform of both the cathode and anode materials. Figure S18b demonstrates the excellent rate performance of the designed sodium-ion battery. At a current density of 0.1 C, the assembled sodium-ion full cell demonstrates a specific capacity of 150 mAh g^−1^ (1 C=100 mA g^-1^), and even under a higher current density of 20 C, the sodium-ion full cell exhibits a reversible specific capacity of 71 mAh g^−1^. Even at high current densities, sodium-ion full batteries still exhibit clearly visible electrochemical charging and discharging platforms (Fig. 3g). As illustrated in Fig. S18c, after 500 cycles at a current density of 5 C, the sodium-ion full cell maintains a capacity retention of 71.4%. To further assess the practical viability of the high-entropy MOFs material, sodium-ion capacitors were assembled using capacitive energy storage-activated carbon as the cathode and high-entropy MOFs material as the anode. In order to obtain the optimum cathode and anode mass ratios, sodium-ion capacitors assembled with different cathode and anode mass ratios were tested for constant current charge and discharge curves (cathode and anode mass ratios from 1:1 to 3:1). As shown in Figs. 3h and S19a, b, the curves for charging and discharging of the assembled sodium-ion capacitors are all linear-like with no obvious voltage plateau, indicating that the rapid interfacial reactions in the sodium-ion capacitors [50]. In addition, the cyclic voltammetric curves of the assembled sodium-ion capacitors were tested as indicated in Fig. S19d. The rectangular shape indicates the existence of two reaction mechanisms in sodium-ion capacitors, namely, Faraday and non-Faraday reactions [51]. As illustrated in Fig. S19c, sodium-ion capacitors with a cathode to anode mass ratio of 2:1 exhibit an optimal combination of power density and energy density. In detail, the sodium-ion capacitor exhibits an energy density of 99.4 Wh kg^−1^ at a power density of 200 W kg^−1^. Even with a 20-fold increase in power density (i.e., 4000 W kg^−1^), it still maintains an energy density of 72.9 Wh kg^−1^. It is worth mentioning that the capacitor exhibits an energy density of 33.3 Wh kg^−1^ at a power density of 20,000 W kg^−1^. To further demonstrate the cycling stability of the assembled sodium-ion capacitors, long cycle tests were carried out at high current density of 1.0 A g^−1^. After 900 charge/discharge cycles, the sodium-ion capacitors exhibited a capacity retention rate of 73.68% (Fig. S19e). To further confirm the absence of sodium metal deposition on the high-entropy MOFs electrode, sodium-ion capacitor cells cycled for 900 cycles were disassembled, and the surface of the high-entropy MOFs electrode was observed (Fig. S20a), revealing no significant deposition of sodium metal. Simultaneously, XPS tests were conducted on the electrode surface (Fig. S20b, c), revealing a distinct peak at 1071.68 eV for Na 1*s*, attributed to Na–O and Na-F bonds, with no characteristic peak of metallic sodium detected. In summary, sodium-ion batteries and sodium-ion capacitors demonstrate excellent electrochemical performance, further confirming the practical value of high-entropy MOFs in high-security fast-charging sodium storage devices.

### SEI Film Analysis

**Fig. 4:**
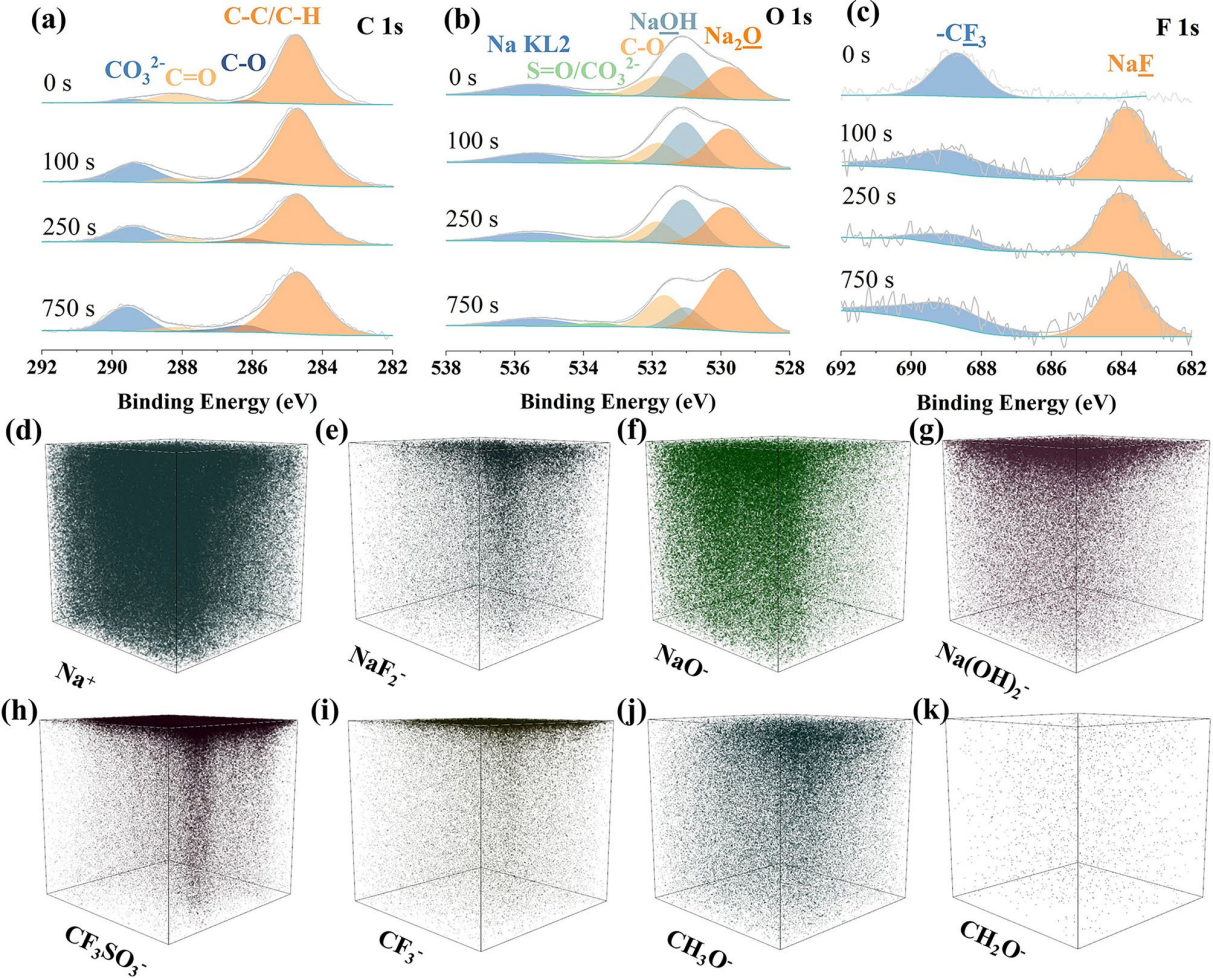
In-depth analysis of SEI components of HEM-2 electrodes. **a–c** High-resolution XPS of C 1*s*, O 1*s*, and F 1*s* spectra with sputtering time for 0, 100, 250, and 750 s, respectively. **d–k** Three-dimensional rendering models of Na^+^, NaF^−^, NaO^−^, Na(OH)_2_^−^, CF_3_SO_3_^−^, CF_3_^−^, CH_3_O^−^, and CH_2_O^−^ for HEM-2 electrodes

To further explore the excellent electrochemical performance of high-entropy MOFs, argon ion sputtering XPS and time-of-flight secondary ion mass spectrometry (TOF–SIMS) were conducted to characterize the composition of the SEI film. Fig. 4a-c, respectively, displays the variation of C 1*s*, O 1*s*, and F 1*s* spectra in the SEI film of HEM-2 electrodes with increasing sputtering time (sputtering rate of 0.02 nm s^−1^). The decomposition of the electrolyte led to the formation of the SEI layer, primarily comprising organic phases, including C–C/C-H (284.8 eV), C-O (286.16 eV), C=O (288.11 eV), -CF_3_ (688.98 eV), O=S=O (533.39 eV), as well as inorganic compounds such as Na_2_O (529.87 eV), NaOH (531.00 eV), Na_2_CO_3_ (289.41 eV), and NaF_2_ (683.86 eV), etc. [52, 53]. From the F 1*s* spectrum, it can be observed that no characteristic peak of NaF was detected before the start of argon ion sputtering. At 100 s of sputtering, a strong peak of sodium fluoride (NaF) was detected, and the peak of -CF_3_ significantly weakened, indicating that NaF replaced -CF_3_ as the main component in the F 1*s* spectrum. The abundant NaF in the SEI membrane facilitates the transport of sodium ions and inhibits electron leakage, thus reducing the risk of sodium metal deposition on the electrode surface at high current densities and improving the safety performance of the battery [53–55]. TOF–SIMS was employed for three-dimensional (3D) surface reconstruction of the high-entropy MOFs electrode surfaces (sputtering time of 1500 s and sputtering depth of 50 nm). Figure 4d-k presents the 3D reconstructions of Na^+^, NaF_2_^-^, NaO^-^, Na(OH)_2_^-^, CF_3_SO_3_^-^, CF_3_^-^, CH_3_O^-^, and CH_2_O^-^. The TOF–SIMS data further confirmed the composition analysis results of the SEI film by XPS. Additionally, from the 3D reconstructions in Fig. 4e, i, it is evident that NaF_2_^-^ is distributed both on the surface and internally of the high-entropy electrodes, while CF_3_^-^ is mainly distributed on the surface, consistent with the XPS results. Meanwhile, CF_3_SO_3_^-^ ions and a small amount of organics derived from electrolyte decomposition, CH_3_O^-^ and CH_2_O^-^, are still detectable on the electrode surface. A significant amount of Na^+^, NaO^-^, and Na(OH)_2_^-^ are detectable on both the surface and internally of the electrode. Similarly, three-dimensional (3D) surface reconstruction was performed on the Mn-MOFs electrode surfaces (with the same sputtering time and depth as the high-entropy MOFs electrodes). Figure S21 shows the 3D reconstructions of Na^+^, NaF_2_^-^, NaO^-^, Na(OH)_2_^-^, CF_3_SO_3_^-^, CF_3_^-^, CH_3_O^-^, and CH_2_O^-^. In particular, the signal intensity of CF_3_SO_3_^-^ in the 3D reconstruction of Mn-MOFs is significantly higher than that in the high-entropy MOFs electrodes, indicating a lesser amount of CF_3_SO_3_^-^ involved in the formation of the SEI film in Mn-MOFs, and thus, the amount of inorganic components produced is also lower. To further validate this conjecture, we compared the signal intensities of CF_3_SO_3_^-^ and inorganic components (NaF_2_^-^, NaO^-^, and Na(OH)_2_^-^) in both MOFs. As shown in Fig. S22, the CF_3_SO_3_^-^ at the electrode of the high-entropy MOFs is significantly less than that of the Mn-MOFs, whereas inorganic component is significantly more than that of the Mn-MOFs. The higher content of the inorganic component facilitates the migration of the sodium ions, which improves the rate performance of the MOFs [55, 56].

### Elemental Effects and Reaction Mechanisms

The progressively decreasing content of Mn elements in high-entropy MOFs shows a large effect on the reversible specific capacity of the material. To further investigate the effect of other metal elements on the electrochemical performance, a number of series of binary MOFs were synthesized. The CV tests were carried out on button cells assembled from different MOFs materials. As shown in Figs. S14d and S23a-d, binary MOFs exhibit similar oxidation peaks compared to Mn-MOFs. It is notable that the reduction peaks are all below 1 V, but they differ in shape and number suggesting that the introduction of different elements may have an effect on the electrochemical properties. In order to further investigate the effect of different elements on the electrochemical performance, cyclic tests were carried out on the MOFs material. As displayed in Figs. S14h and S23e-h, the first Coulombic efficiency and reversible specific capacity of the binary MOFs are significantly improved compared to those of the MOFs. In particular, the introduction of Ni and Cu significantly improved the first Coulombic efficiency, and the introduction of Co and Ni greatly enhanced the reversible specific capacity, while the introduction of Zn improved both the first Coulombic efficiency and the reversible specific capacity, but the effect was not obvious. Long cycle tests at 5 A g^−1^ are shown in Fig. S23i-l. The reversible specific capacity of individual Mn-MOFs is poor as soon as possible, but it shows excellent cycling stability (Fig. 3e). The terrible thing is that the introduction of Co, Ni, and Cu elements also presents poor cyclic stability despite the reversible increase in specific capacity. Furthermore, although the cycling retention of binary MnZn-MOFs is inferior to that of Mn-MOFs, it is much better than the cycling retention of the other three binary MOFs. This indicates that the introduction of Zn elements improves the stability of MOFs materials more than the introduction of other metal elements.

**Fig. 5:**
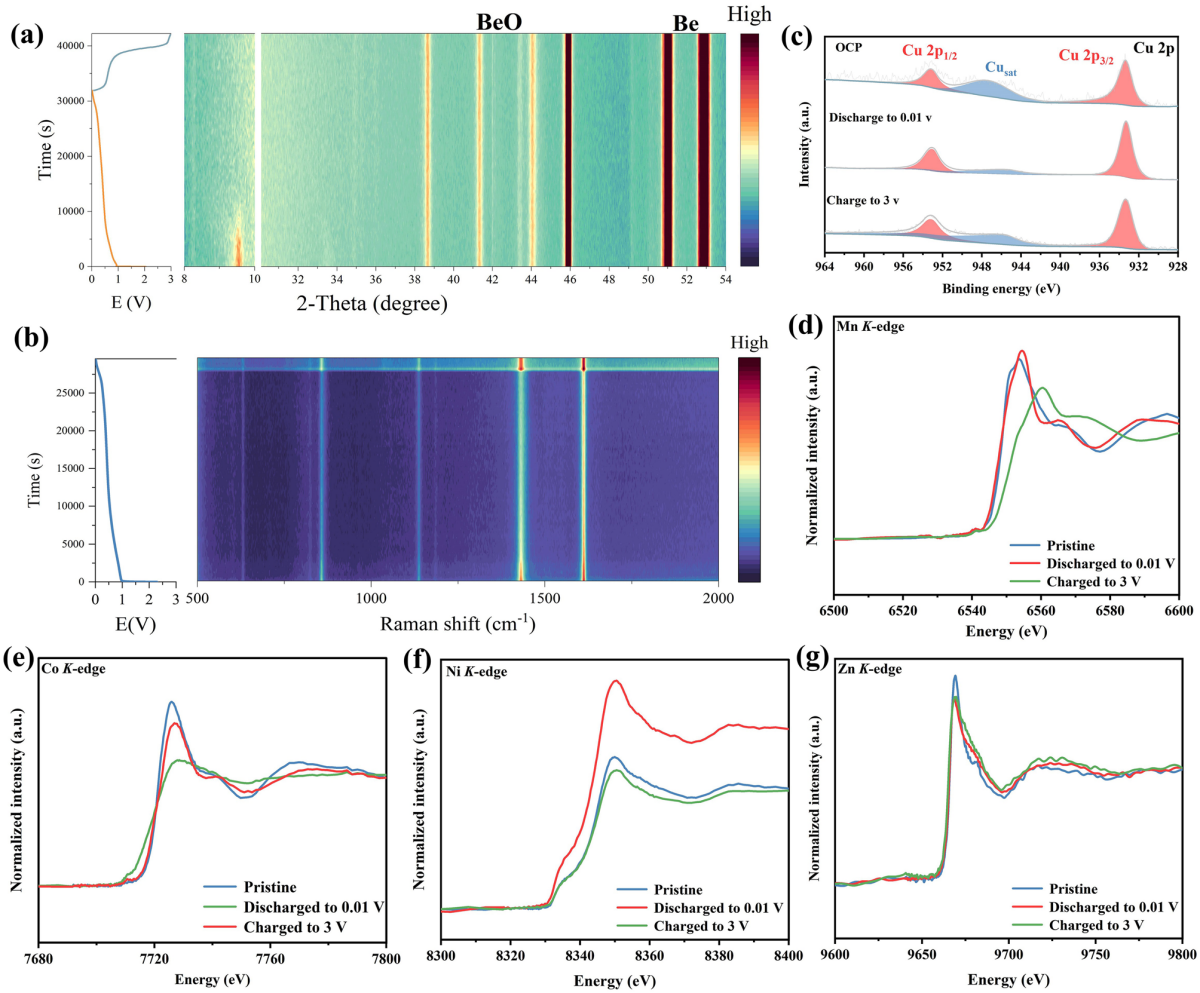
Charging and discharging mechanism.** a** In situ XRD of HEM-2 electrode. **b** In situ Raman spectrum of HEM-2 electrode. **c** XPS of Cu 2*p* in different charging and discharging states. **d–g** Mn K-edge, Co K-edge, Ni K-edge, and Zn K-edge of HEM-2 electrode in different charging and discharging states

The introduction of different elements can affect the electrochemical properties differently; however, it is not clear whether the introduced metallic elements participate in the sodium storage reaction. In order to determine the sodium storage mechanism of MOFs, the in situ and ex situ characterization of HEM-2 electrodes was analyzed during sodium-ion insertion and detachment. Figure 5a illustrates the discharge–charge curves and corresponding XRD data for HEM-2 at 0.1 A g^−1^ in the first cycle, showing the transformation of the HEM-2 material into an amorphous form after the first sodiation/desodiation process. In addition, no peaks of metal monomers were observed. The evolution of structural vibrational modes of the HEM-2 electrode during discharge processes was monitored through in situ Raman measurements (Fig. 5b). As sodium ions are inserted into the bulk phase of high-entropy MOFs, the vibrational intensity of characteristic peaks of the HEM-2 material changes but does not disappear. Additionally, no new characteristic peaks were detected in the Raman spectra. To further investigate the charge compensation mechanism of high-entropy MOFs, ex situ XPS and synchrotron radiation techniques were utilized to study the valence state evolution of metal elements in high-entropy MOFs. In order to exclude the influence of the SEI layer, the XPS spectra of the metal elements on the electrode material were measured after an argon ion beam sputter etching treatment (Figs. S24a-d and Fig. 5c). When discharged to 0.01 V, the 2*p*_3/2_ orbitals of Co and Ni shift to lower potentials, indicating a reduction in the valence states of Co and Ni. Upon charging to 3 V, the 2*p*_3/2_ orbitals of Co and Ni exhibit chemical shifts similar to their initial states, demonstrating the reversible changes of Co and Ni during charge and discharge processes. During charge and discharge, there is no significant shift observed in the 2*p*_3/2_ orbitals of Cu and Zn elements, indicating that Cu and Zn, as inert elements, do not participate in the charge compensation mechanism. Due to the insignificant chemical shift in the 2*p*_3/2_ orbitals of Mn elements at different valence states, in ex situ XPS, the 2*p*_3/2_ orbitals of Mn show no significant movement, making it difficult to accurately determine whether Mn elements are involved in the electrochemical reactions. To accurately elucidate the evolution of metal oxidation states in high-entropy MOFs during charge and discharge, ex situ synchrotron radiation techniques were employed. As shown in Fig. 5d, no significant shift in the K-edge of Mn elements in high-entropy MOFs was observed when discharged to 0.01 V, indicating that the oxidation state of Mn elements does not decrease during discharge. Upon charging to 3 V, the oxidation state of Mn elements increases, consistent with the evolution of Mn oxidation states in Mn-MOFs (Fig. S25). Figure 5e, f shows the data for Co and Ni K-edges. When discharged to 0.01 V, the oxidation states of Co and Ni elements decrease, and when charged to 3 V, the Co and Ni K-edges almost overlap with the initial states, indicating the involvement of Co and Ni in the charge compensation mechanism, consistent with the XPS data. During charge and discharge, there is no significant change observed in the K-edge of Zn elements, suggesting that Zn elements do not participate in the charge compensation mechanism, consistent with the XPS data (Fig. 5g). Since copper foil was used as collector in the electrodes of the high-entropy MOFs, K-edge data of copper were not tested. In conclusion, the characterization results confirm that high-entropy MOFs undergo redox reactions of organic ligands as well as charge compensation of some metal elements. Among them, Mn, Co, and Ni elements are involved in the charge compensation mechanism, while Cu and Zn elements are not.

## Conclusion

In summary, leveraging the tunable metal active site characteristics of metal–organic frameworks, we designed two-dimensional high-entropy metal–organic frameworks (HE-MOFs) with rapid charge–discharge capabilities and achieved precise control over the platform capacity/voltage of the electrode materials. Density functional theory calculations and electrochemical characterizations confirm that the high-entropy effect helps to reduce the energy barrier for sodium-ion migration, thus enhancing the ion migration rate. Moreover, characterization techniques such as synchrotron X-ray absorption spectroscopy validate the charge compensation mechanism of high-entropy MOFs and the influence of elemental effects on electrochemical performance. The two-dimensional high-entropy MOF material exhibits ultra-fast sodium-ion storage capacity and high safety performance, demonstrating practical value in the fields of sodium-ion batteries and capacitors. This work provides new insights into the direct application of thousands of non-conductive MOFs to energy storage electrode materials and opens up new avenues for designing high-safety fast-charging devices.

## Supplementary Information

Below is the link to the electronic supplementary material.

Supplementary file1 (DOC 49909 KB)
