# Supplementary material for: Tunable Platform Capacity of Metal–Organic Frameworks via High-Entropy Strategy for Ultra-Fast Sodium Storage
Source: Nanomicro Lett. 2025 Mar 26;17:201. doi: 10.1007/s40820-025-01706-3 (PMC11947337; doi:10.1007/s40820-025-01706-3)
Supplement: Supplementary file 1 — Supplementary file1 (DOC 49909 KB) [file 40820_2025_1706_MOESM1_ESM.doc]

Supporting Information for

**Tunable Platform Capacity of Metal-Organic Frameworks via High Entropy Strategy for Ultra-Fast** **Sodium Storage**

Shusheng Tao^1,†^, Ziwei Cao^1,†^, Xuhuan Xiao^1,2^, Zirui Song^1,3^, Dengyi Xiong^1^, Ye Tian^1^, Wentao Deng^1^, Youcai Liu^1^, Hongshuai Hou^1^, Guoqiang Zou^1^* and Xiaobo Ji^1^

^1^College of Chemistry and Chemical Engineering, Central South University, Changsha 410083, P. R. China

^2^Department of Chemistry, University College London, London, WC1H 0AJ, UK

^3^Department of Materials, University of Oxford, Oxford, OX1 3PH UK

**^†^**Shusheng Tao and Ziwei Caocontributed equally to this work.

*Corresponding author. E-mail: [gq-zou@csu.edu.cn](mailto:gq-zou@csu.edu.cn) (Guoqiang Zou)

**S1.1 Theoretical calculations**

This study used the Cambridge Serial Total Energy Package (CASTEP) software for Density functional theory (DFT) calculations. The generalised gradient approximation (GGA) of the Perdew-Burke-Ernzerhof (PBE) generalisation was employed to calculate the electron exchange correlation potentials and the ultra-soft pseudopotentials were used. The kinetic energy cutoff of the plane-wave basis group is set to 450 eV. the Brillouin zone integral is set to 4×4×3. Single Mn-MOFs and high-entropy MOFs models are constructed, where Mn, Co, Ni, Cu, and Zn occupy the same sites in the high-entropy MOFs model. The binding energies of Co, Ni, Cu, and Zn replacing the sites of Mn elements in Mn-MOFs were calculated and defined as E_form_=[E(M/Mn-MOFs)-aE(C)-bE(O)-cE(H)-dE(Mn)-E(M)]/(a+b+c+d+1), where “M” is the above four metallic elements (M=Co, Ni, Cu, Zn). The diffusion energy barriers of Na^+^ were tested using synchronous transit methods, including linear synchronous transition (LST) and quadratic synchronous transition (QST). The convergence tolerances were set as 2×10^-5^ eV per atom for energy, 2×10^-3^ Å for maximum displacement, and 0.05 eV/Å for maximum force. The adsorption of Na^+^ by the materials was investigated, the electrochemical properties of the materials were demonstrated, and the adsorption energy of the materials on Na^+^ was calculated, defined as ΔE=E(slab+Na^+^)-E(slab)-E(Na^+^). All of the structures were fully optimized and relaxed to the ground state.

**S1.2 Material synthesis methods**

Preparation of Mn-MOFs: All the raw materials belong to analytical grade and have been used directly. Mn-MOFs were synthesized based on previously reported paper. To synthesize Mn-MOF, 4.062 g (16.5 mmol) Mn (CH_3_COO)_2_·4H_2_O and 4.752 g sodium benzoate (33 mmol) were dissolved in 60 mL dimethylformamide (DMF) and stirred for 10 h at room temperature. Then 5.502 g (33 mmol) terephthalic acid (PTA) was added to above solution to stir 1 h. After that the solution was transferred into a 100 mL Teflon lined stainless steel autoclave and reacted at 180 °C for 10 h. After a slow cooling down to room temperature, the white precipitate was collected by centrifuge and washed with ethanol and distilled water three times, respectively. Then the obtained Mn-MOF was dried at 80 °C in a vacuum oven.

Preparation of HEM-1/2/3: The HEM-1 was synthesized with the similar method with the 40% Mn (CH_3_COO)_2_·4H_2_O and 15% Co(CH_3_COO)_2_·4H_2_O、15% Zn(CH_3_COO)_2_、15% Ni(CH_3_COO)_2_·4H_2_O、15% Cu(CH_3_COO)_2_·H_2_O. The HEM-2 was synthesized with the similar method with the 60% Mn (CH_3_COO)_2_·4H_2_O and 10% Co(CH_3_COO)_2_·4H_2_O、10% Zn(CH_3_COO)_2_、10% Ni(CH_3_COO)_2_·4H_2_O、10% Cu(CH_3_COO)_2_·H_2_O. The HEM-3 was synthesized with the similar method with the 80% Mn (CH_3_COO)_2_·4H_2_O and 5% Co(CH_3_COO)_2_·4H_2_O、5% Zn(CH_3_COO)_2_、5% Ni(CH_3_COO)_2_·4H_2_O、5% Cu(CH_3_COO)_2_·H_2_O. The amounts of metal salts and organic ligands used were consistent with those of Mn-MOFs.

Preparation of bimetallic MOFs: The MnCo-MOFs was synthesized with the similar method with the 60% Mn (CH_3_COO)_2_·4H_2_O and 40% Co(CH_3_COO)_2_·4H_2_O. The MnNi-MOFs was synthesized with the similar method with the 60% Mn (CH_3_COO)_2_·4H_2_O and 40% Ni(CH_3_COO)_2_·4H_2_O. The MnCu-MOFs was synthesized with the similar method with the 60% Mn (CH_3_COO)_2_·4H_2_O and 40% Cu(CH_3_COO)_2_·H_2_O. The MnZn-MOFs was synthesized with the similar method with the 60% Mn (CH_3_COO)_2_·4H_2_O and 40% Zn(CH_3_COO)_2_. The amounts of metal salts and organic ligands used were consistent with those of Mn-MOFs.

**S1.3 Materials characterization**

The microscopic morphology of Mn-MOFs and high-entropy MOFs was observed by scanning electron microscopy (SEM, Hitachi S-4800) and 200 kV field emission transmission electron microscopy (TEM, FEI talos f200s). Energy dispersive spectroscopy (EDS) was applied to map the distribution of various elements.The proportion of metallic elements in the MOFs materials was determined by inductively coupled plasma optical emission spectroscopy (ICP-OES). X-ray diffraction (XRD) patterns and in situ XRD were recorded using a Rigaku Ultima IV X-ray diffractometer and Cu Kα radiation at 1.5406 Å. Fourier transform infrared spectroscopy (FTIR, PerkinElmer SPECTRUM 100) and Raman spectroscopy (DXR, Thermo-Fisher Scientific, 532 nm) were used to test the MOFs materials. Time-of-flight secondary ion mass spectrometry (TOF-SIMS, PHI NanoTOFII) was used for SEI membrane composition. The Mn L, Co L, Ni L and Cu L side spectra were tested in total electron yield (TEY) mode at the U19 beamline of the National Synchrotron Radiation Laboratory (Hefei, China). The X-ray absorption fine structure (XAFS) spectra of Mn K, Co K, Ni K and Zn K were collected in transmission mode on Table XAFS-500A (Specreation Instruments Co., Ltd.) at 30 kV and 25 mA. The Si (440), Si(533), Si(551), Si(733) spherically bent crystal analyzers with a radius of curvature of 500 mm were used for Mn, Co, Ni and Zn, respectively. Before test, the powder samples were grinded and tableted into slices with diameter of 12.7 mm. Hard XAS data on the Mn K, Co K, Ni K, Cu K, and Zn K sides were recorded in transmission mode at room temperature at the Shanghai Synchrotron Radiation Facility (SHSRF) station BL11B beamline. A Si (111) bicrystal monochromator was used at the station.

**S1.4 Electrochemical measurements**

To prepare the MOFs electrodes, the MOFs material was mixed with conductive carbon black and sodium hydroxycellulose (CMC) binder with a MOFs/conductive carbon black/CMC weight ratio of 7:2:1 in a deionised water solvent. The paste was then applied to the copper foil and dried under vacuum at 80°C overnight. To prepare the cathode, NaVPO_4_ or activated carbon is mixed with conductive carbon black and polyvinylidene fluoride (PVDF) binder with an active substance/conductive carbon black/PVDF weight ratio of 8:1:1 in N-methyl-2-pyrrolidone solvent. The slurry is then applied to the aluminium foil and dried overnight at 120°C under vacuum to remove residual solvent. The electrolyte was 1.0 mol L^−1^ NaCF_3_SO_3_ in DIGLYME=100 Vol%. The electrochemical performance of CR2016 coin type batteries was investigated with sodium metal as counter electrode. The full sodium-ion battery and sodium-ion capacitor require the same diaphragm and electrolyte as the half-cell described above. The assembly of the sodium ion capacitor requires the pre-sodiumation of high-entropy MOFs electrodes. The pre-sodiumation method is an electrochemical method, and the specific steps are to discharge the high entropy MOFs to 0.01 V after five cycles in a half-cell with a current density of 0.1 A g^-1^. The half-cell is disassembled to take out the electrodes of pre-sodiumed high entropy MOFs, and then the activated carbon is used for the assembly of sodium-ion capacitors. Rate and cycle data were obtained using the Landt CT2001A test system. Electrostatic current discharge-charge data and CV data were obtained by Autolab and electrochemical workstations (Shanghai Chenhua, China).

The formulas for energy density (E, Wh kg^-1^) and power density (P, W kg^-1^) of sodium-ion capacitors are in agreement with those reported in the literature and were calculated from formulas based on GCD measurements. The formulas are calculated as follows：

$$\begin{matrix} C=It/\Delta Vm \\ E=\frac{C(V_{2}^{2}-V_{1}^{2})}{2\times3.6} \\ P=\frac{3600\times E}{t} \end{matrix}$$

Where t is the discharge time (s), I is the discharge current (A), ΔV represent the potential change (V), m is the total mass of both anode and cathode active materials, V_2_ and V_1_ is the initial and Final discharge potentials (V).

**S2 Supplementary Figures and Tables**

**
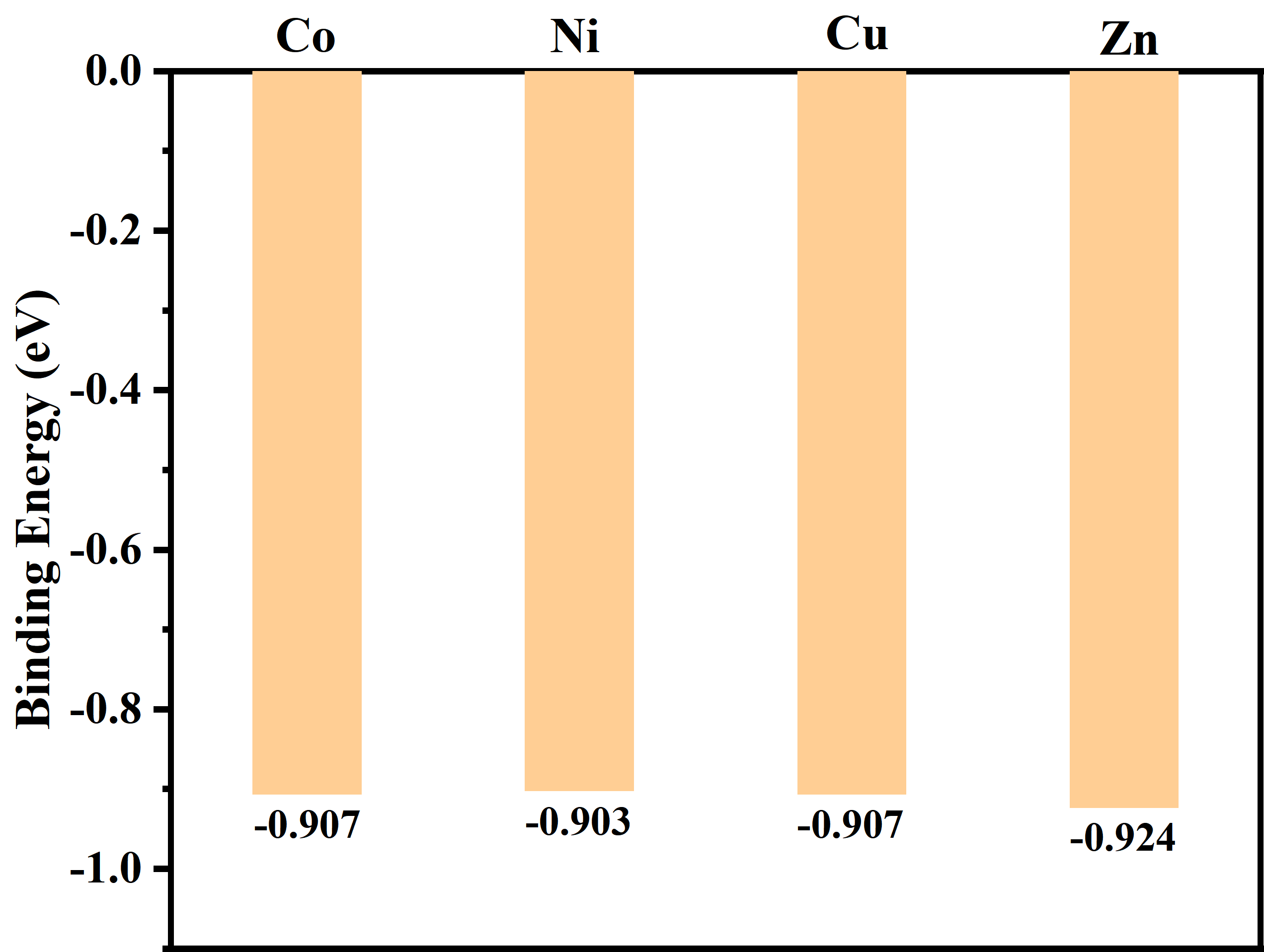
**

**Fig. S1** Binding energy of the Mn site in Mn-MOFs substituted by different elements (Co, Ni, Cu, Zn)

**
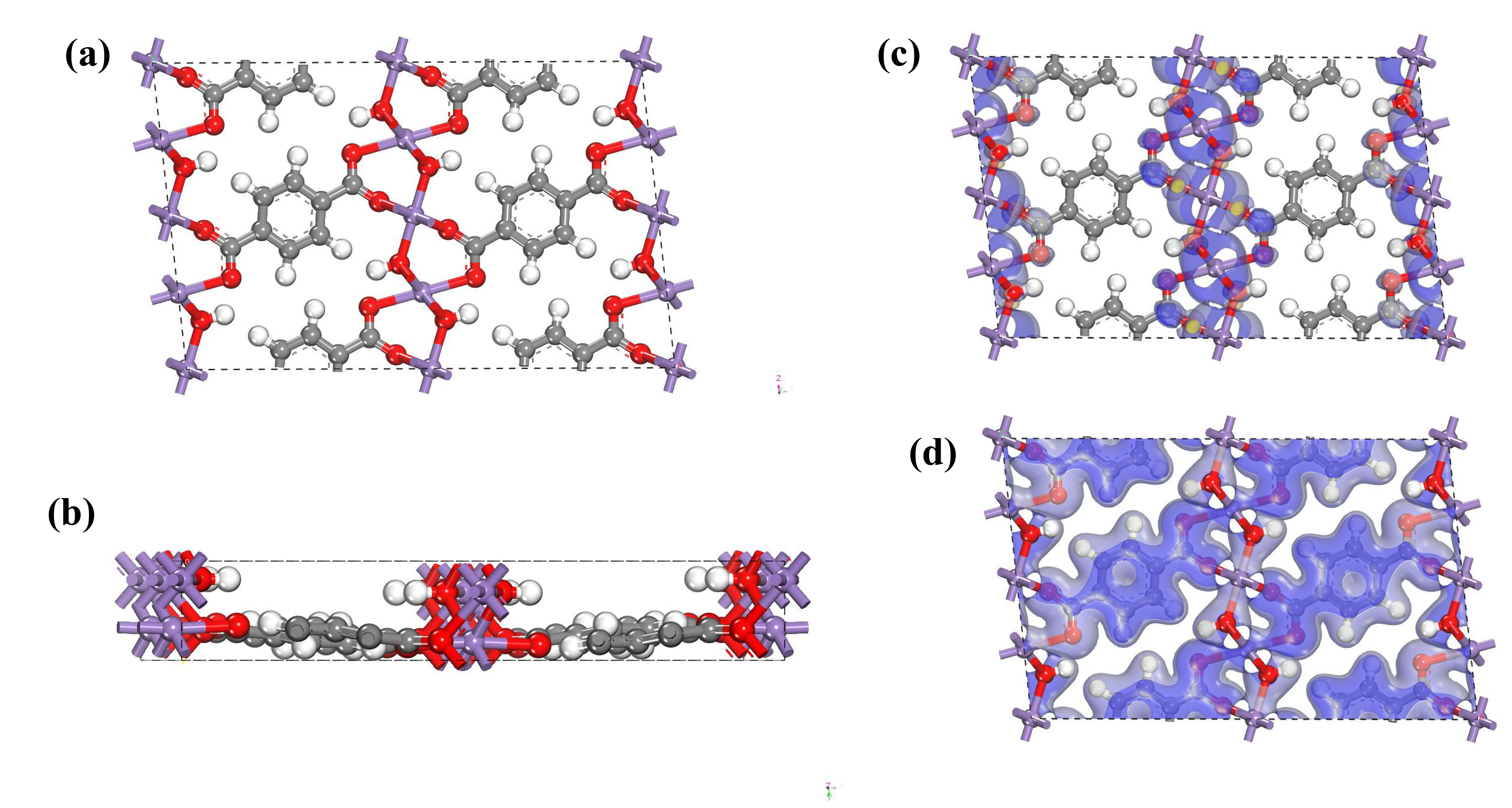
**

**Fig. S2 a.** Top view of crystal structure, **b.** Main view of crystal structure, **c.** Spin density, **d.** Total density of Mn-MOFs
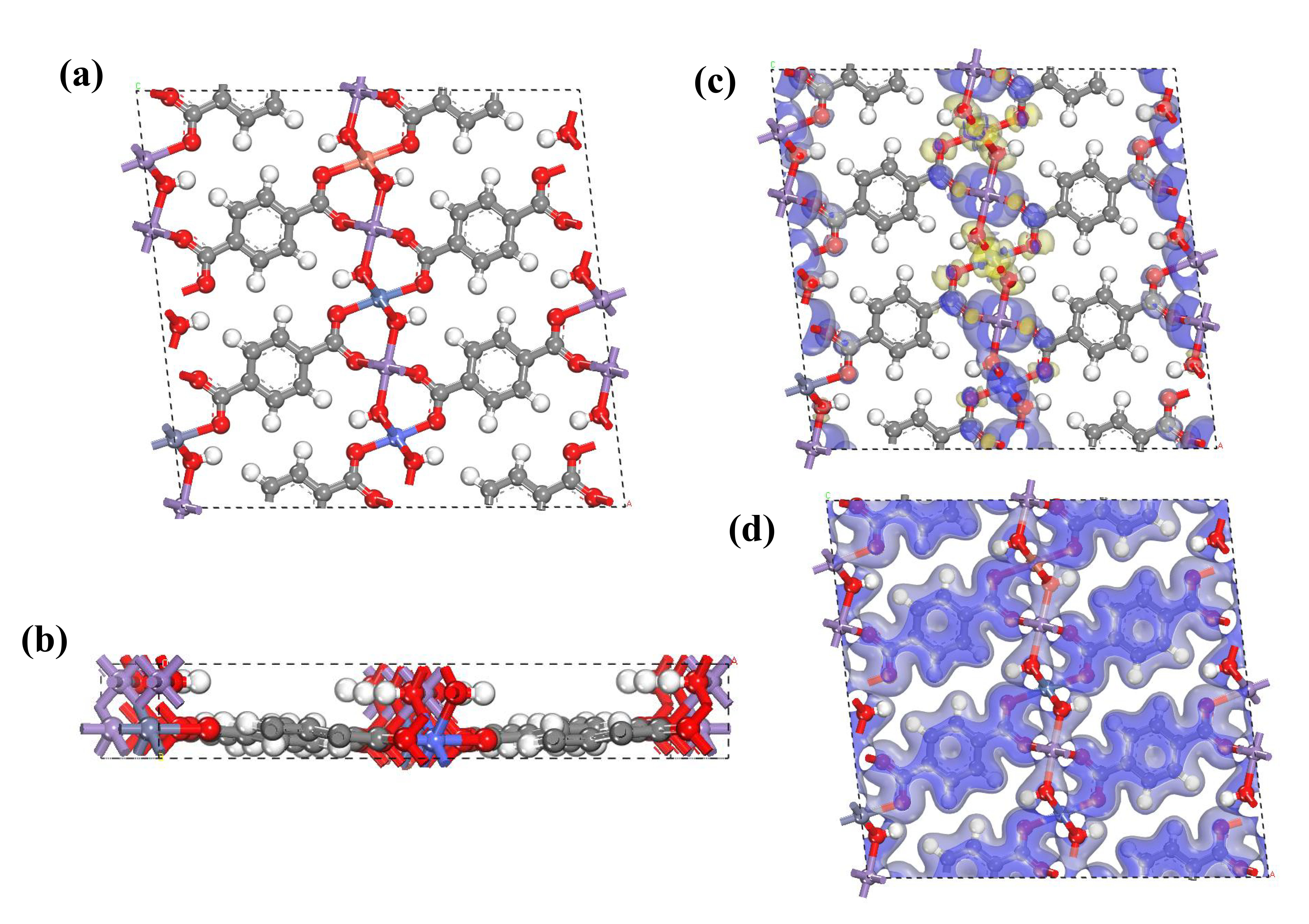


**Fig. S3 a.** Top view of crystal structure, **b.** Main view of crystal structure, **c.** Spin density, **d.** Total density of high entropy MOFs (Elemental Mn at 80 per cent)


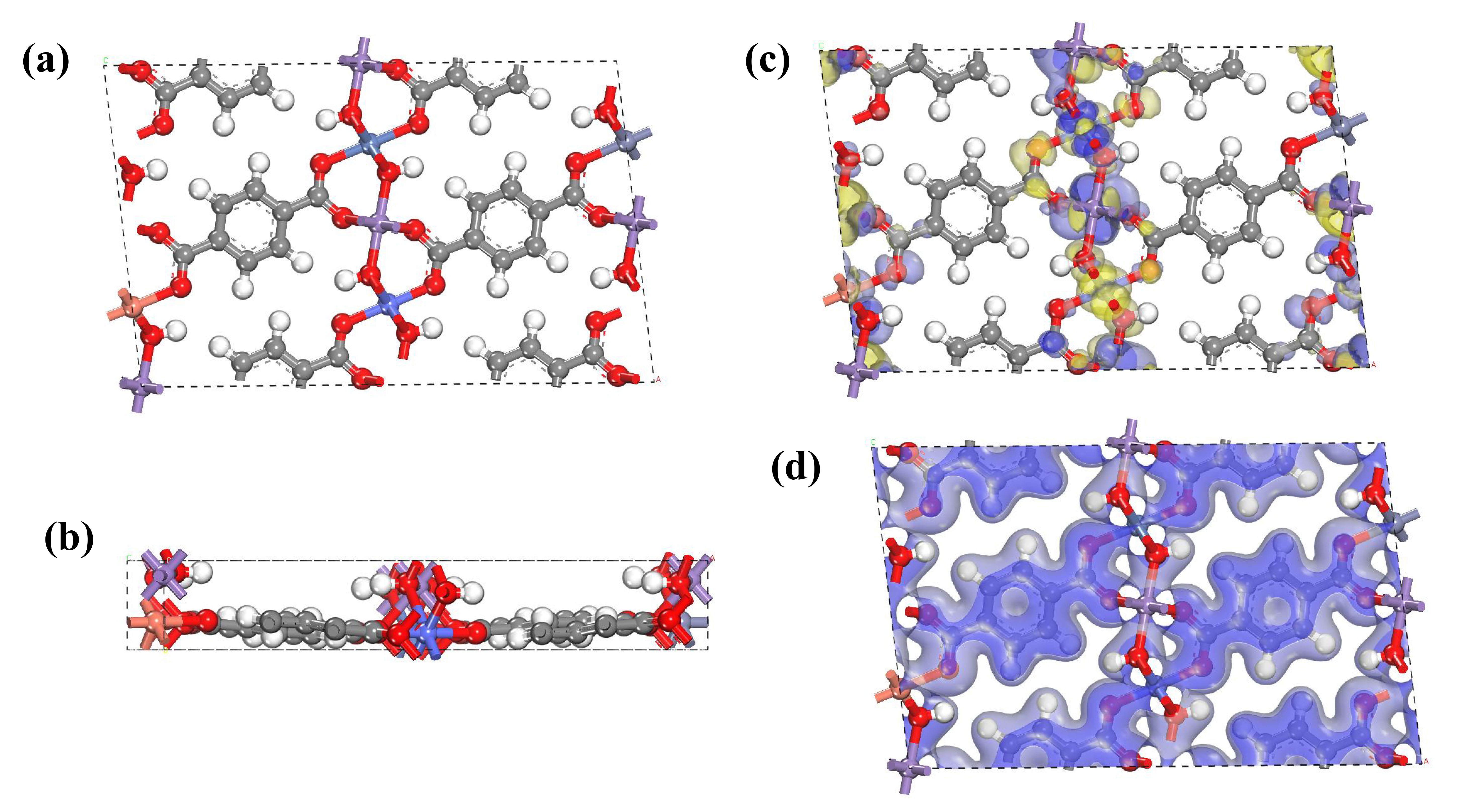


**Fig. S4 a.** Top view of crystal structure, **b.** Main view of crystal structure, **c.** Spin density, **d.** Total density of high entropy MOFs (Elemental Mn at 40 per cent)

**
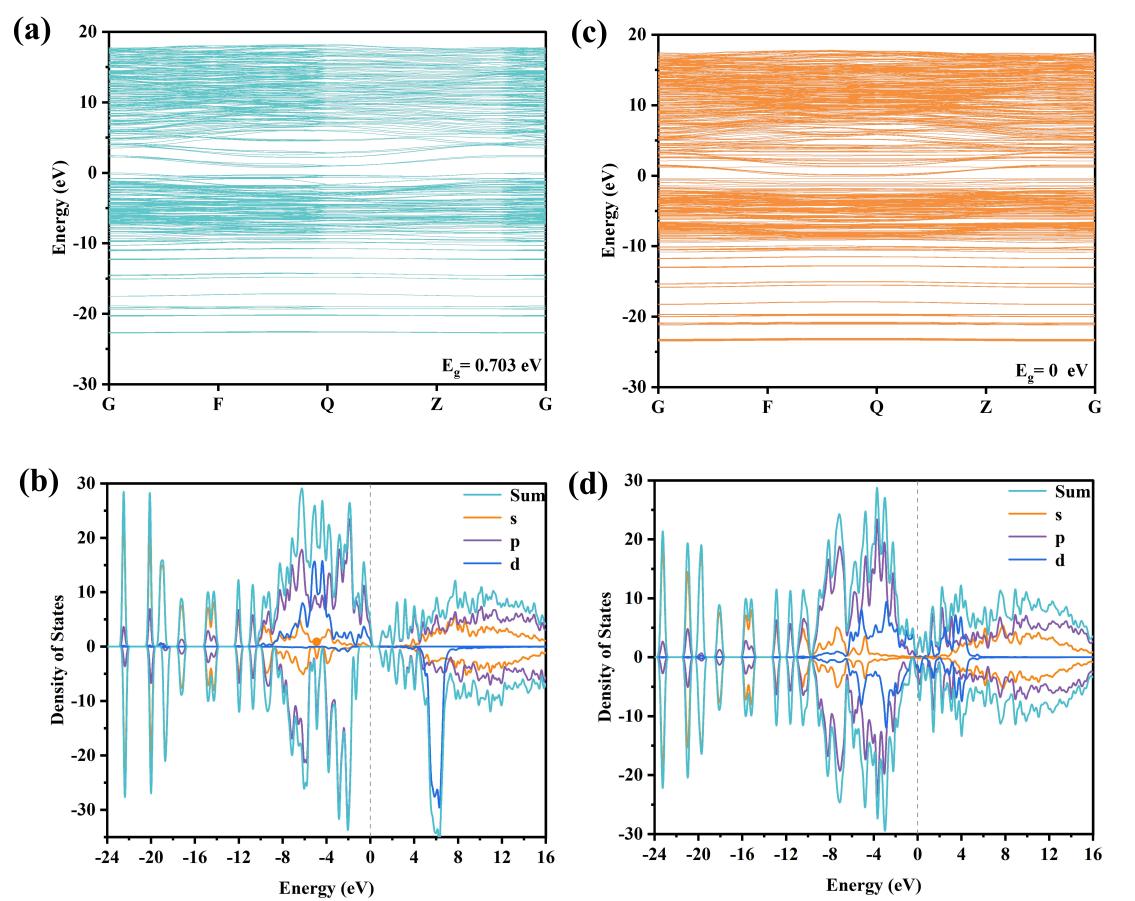
**

**Fig. S5** Energy band gap and density of states of **a, b** Mn-MOFs, **c, d** High entropy MOFs (Elemental Mn at 60 per cent)

**
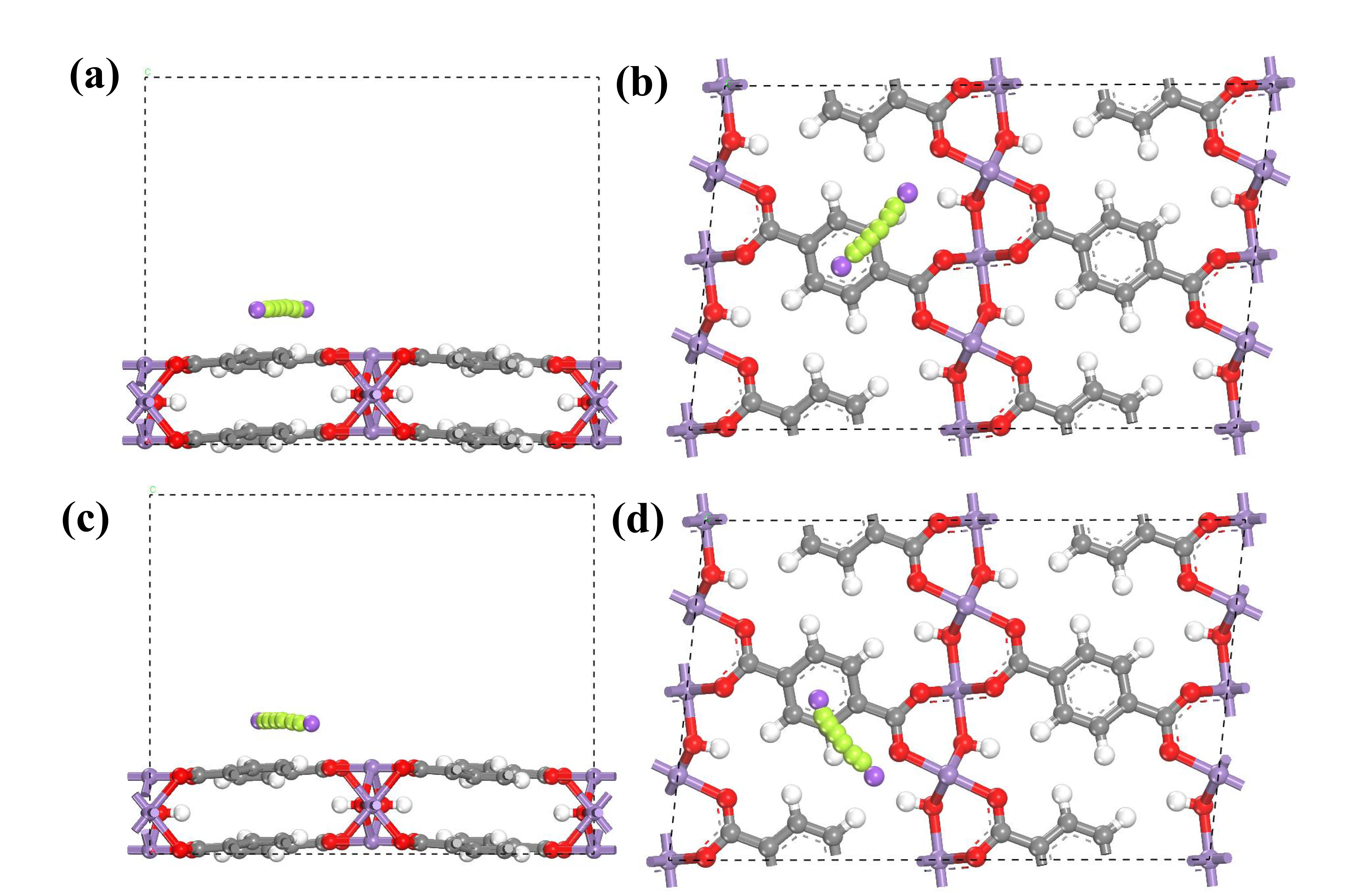
**

**Fig. S6** Sodium ion migration pathway of high-entropy MOFs (Mn content of 60%). **a.** Main view and **b.** Top view of sodium ion migration from near active site 2 to near active site 1. **c.** Main view and **d.** Top view of sodium ion migration from near active site 2 to near active site 3

**
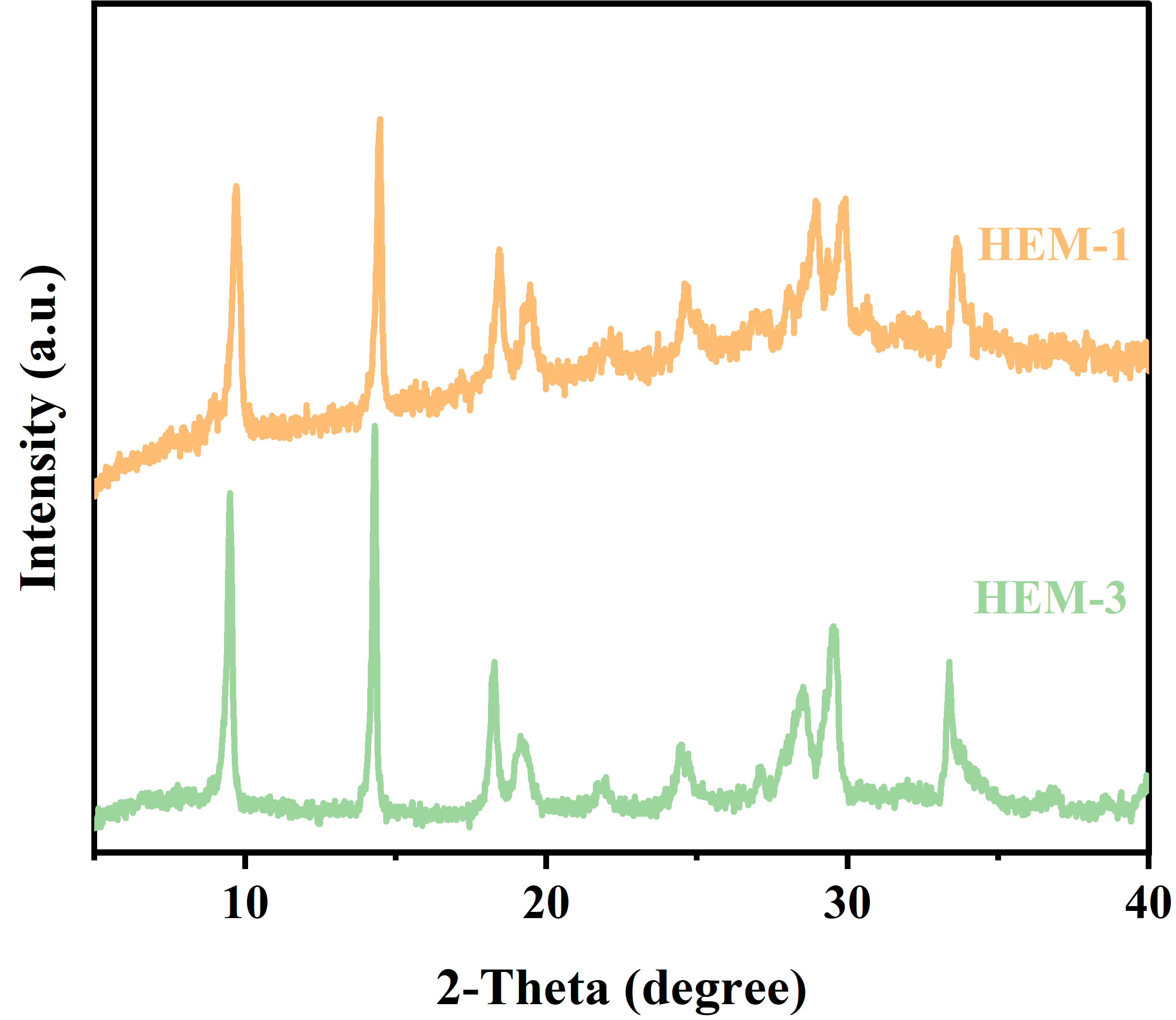
**

**Fig. S7** XRD patterns of high-entropy MOFs materials

**
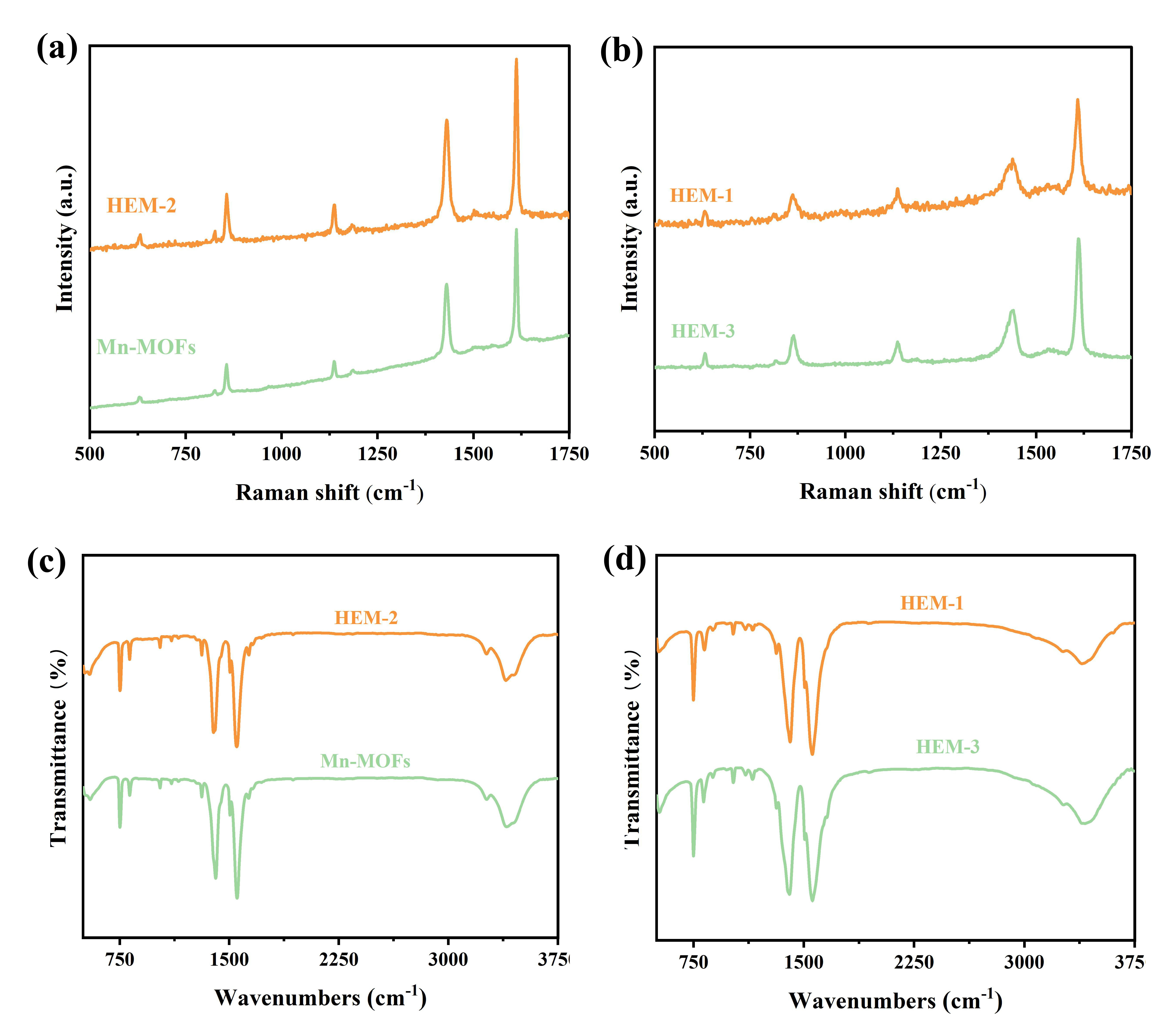
**

**Fig. S8 a, b.** Raman spectrum and **c, d.** FTIR spectra of HEM-1/2/3 and Mn-MOFs


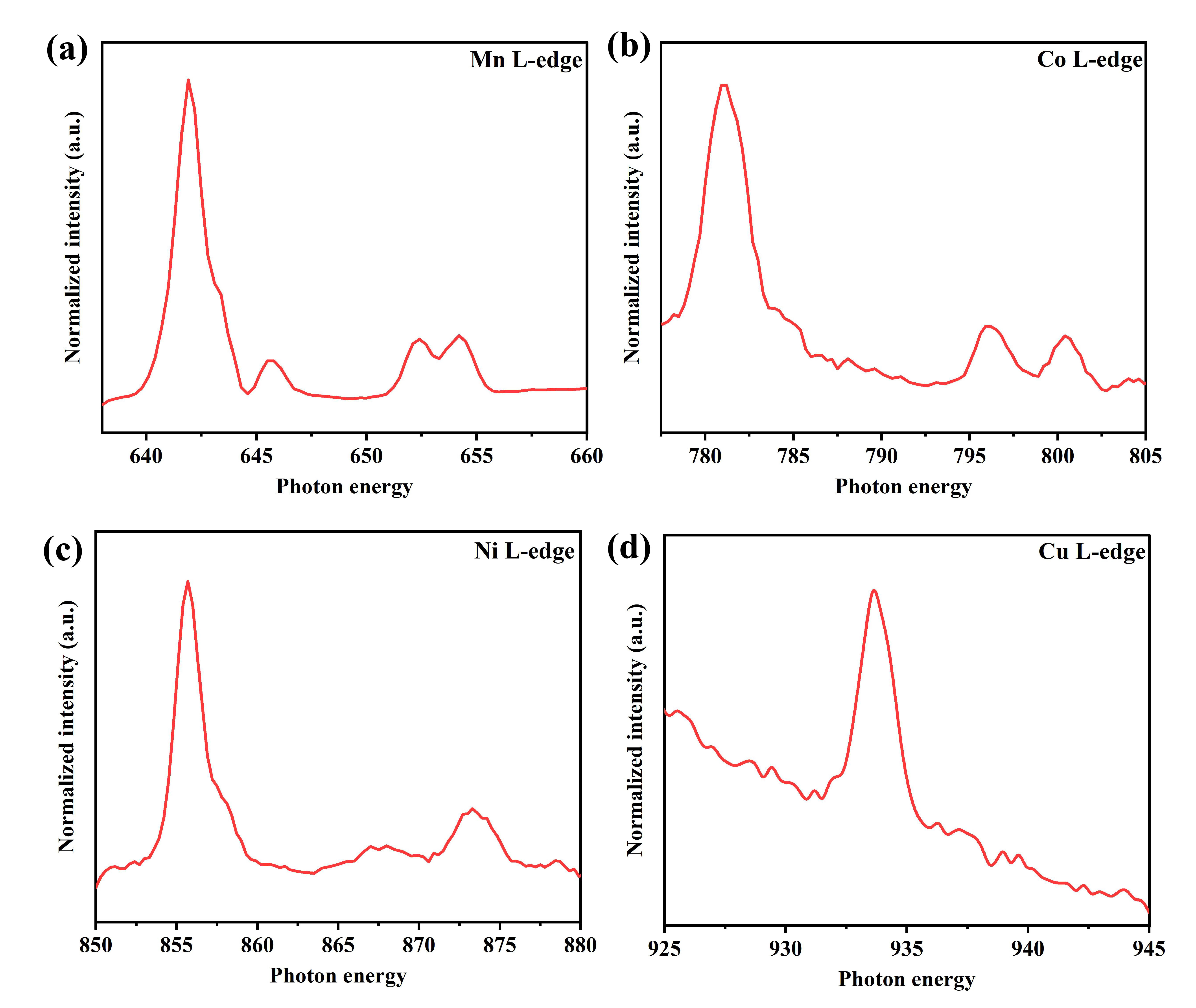


**Fig. S9 a-d** Soft X-ray of synchronous radiation of Mn L-edge, Co L-edge, Ni L-edge and Cu L-edge of HEM-2, respectively


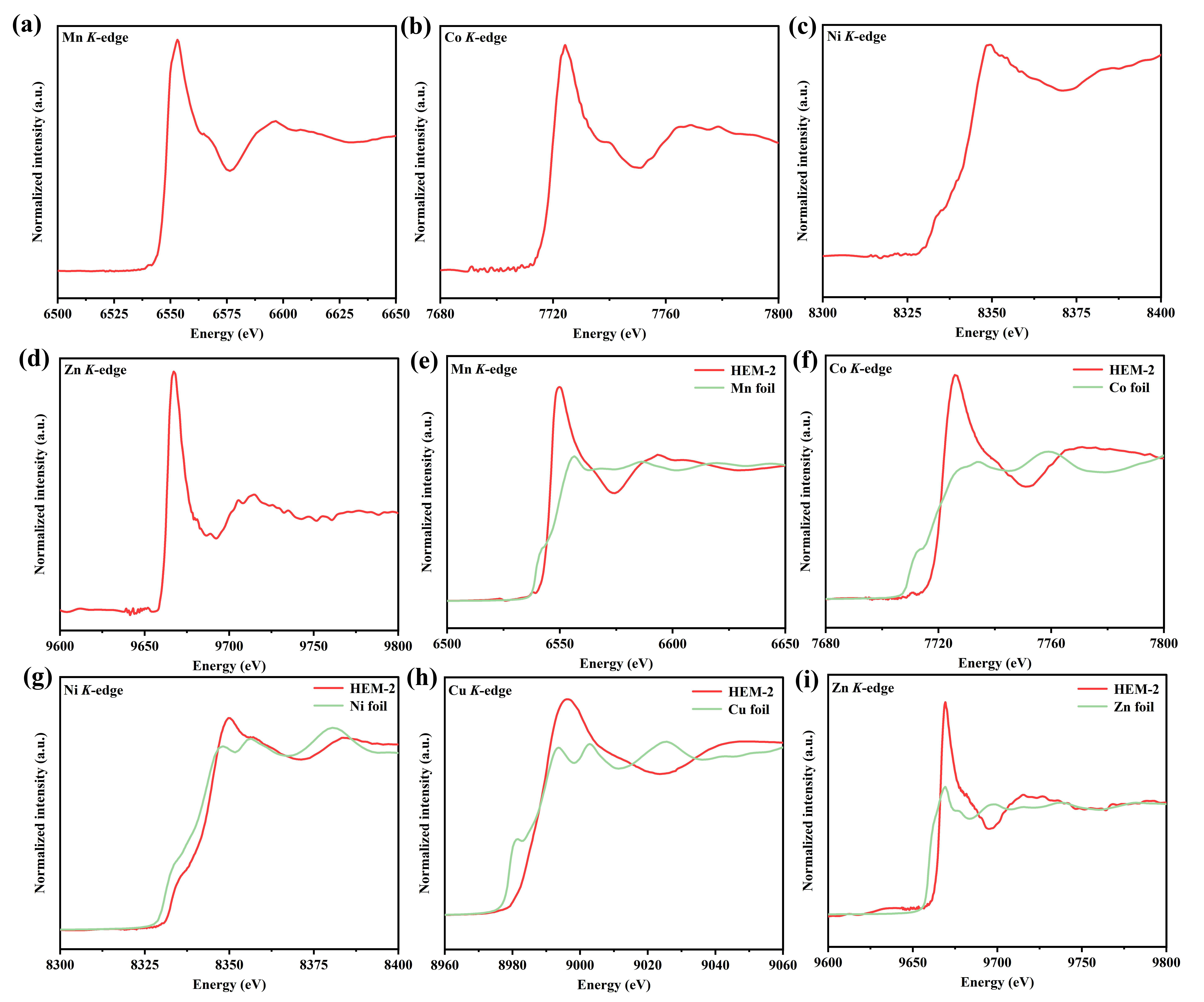


**Fig. S10** K-edge table XAS spectra of **a.** Mn k-edge, **b.** Co-edge, **c.** Ni-edge, **d.** Zn k-edge of HEM-2. K-edge HXAS spectra of **e.** Mn k-edge, **f.** Co-edge, **g.** Ni-edge, **h.** Cu k-edge, **i.** Zn k-edge of HEM-2

**
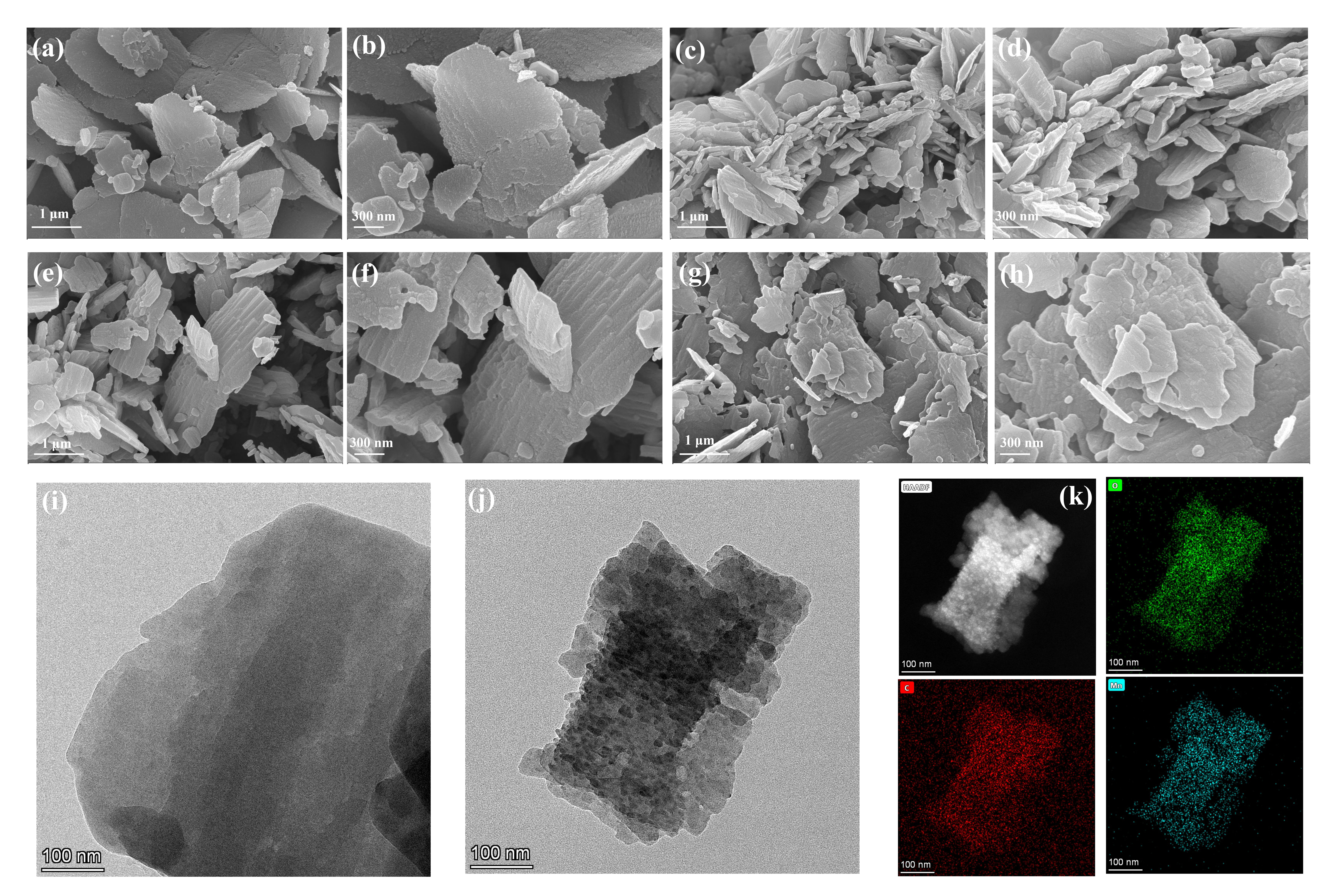
**

**Fig. S11** SEM images of **a, b.** HEM-1, **c, d.** HEM-2, **e, f.** HEM-3, and **g, h.** Mn-MOFs. TEM image of **i.** HEM-2, and **j.** Mn-MOFs. **k.** Elemental mappings (O, C and Mn) of Mn-MOFs


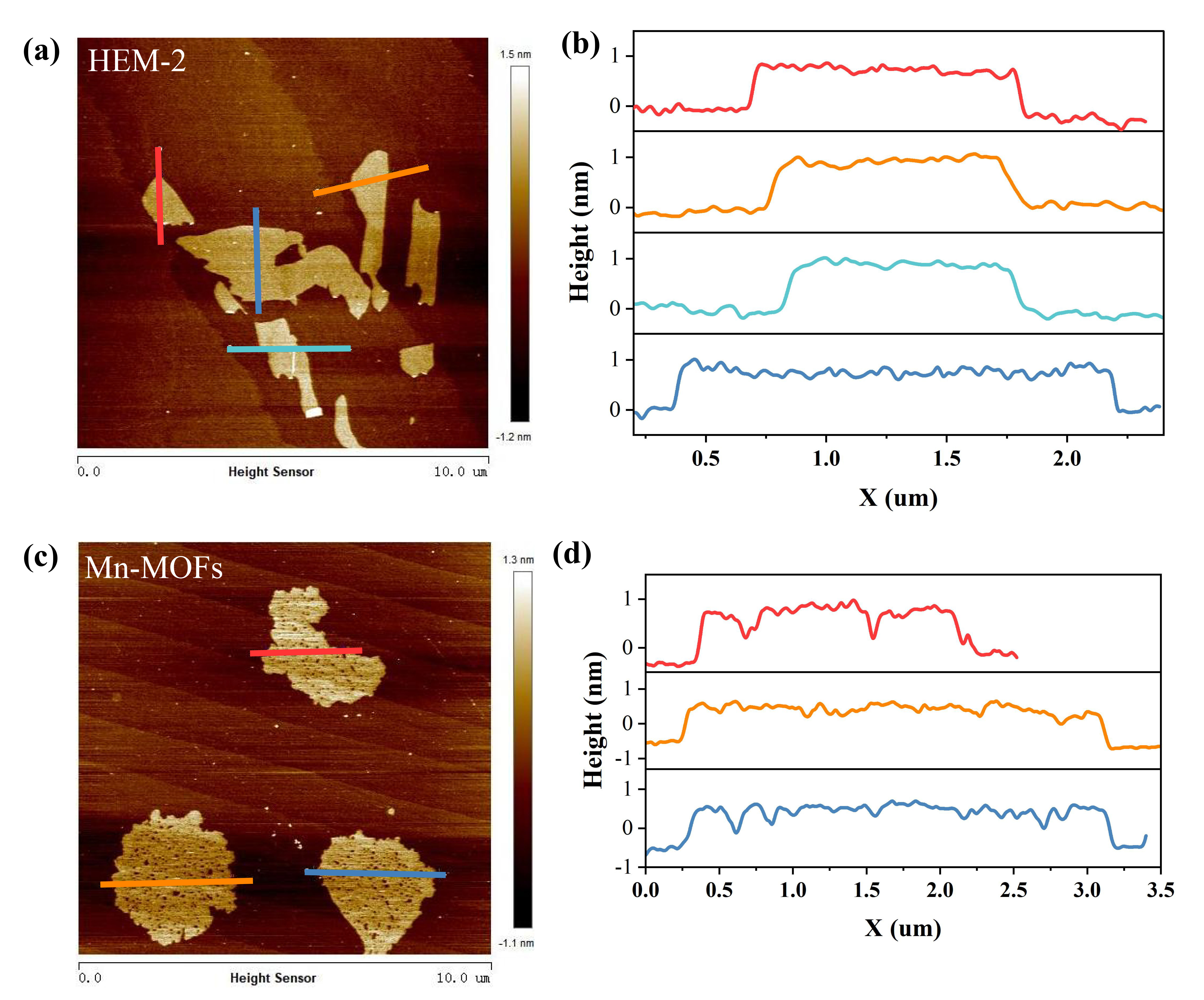


**Fig. S12** Estimated thickness of the nanosheets **a, b.** HEM-2. **c, d.** Mn-MOFs


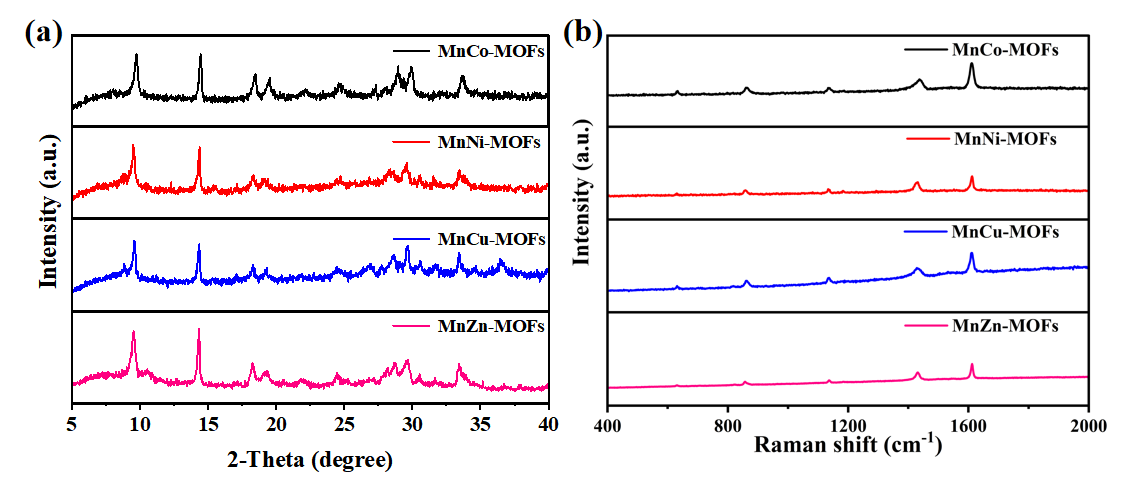


**Fig. S13** **a.** XRD patterns and **b.** Raman spectrum of the bimetallic MOFs materials


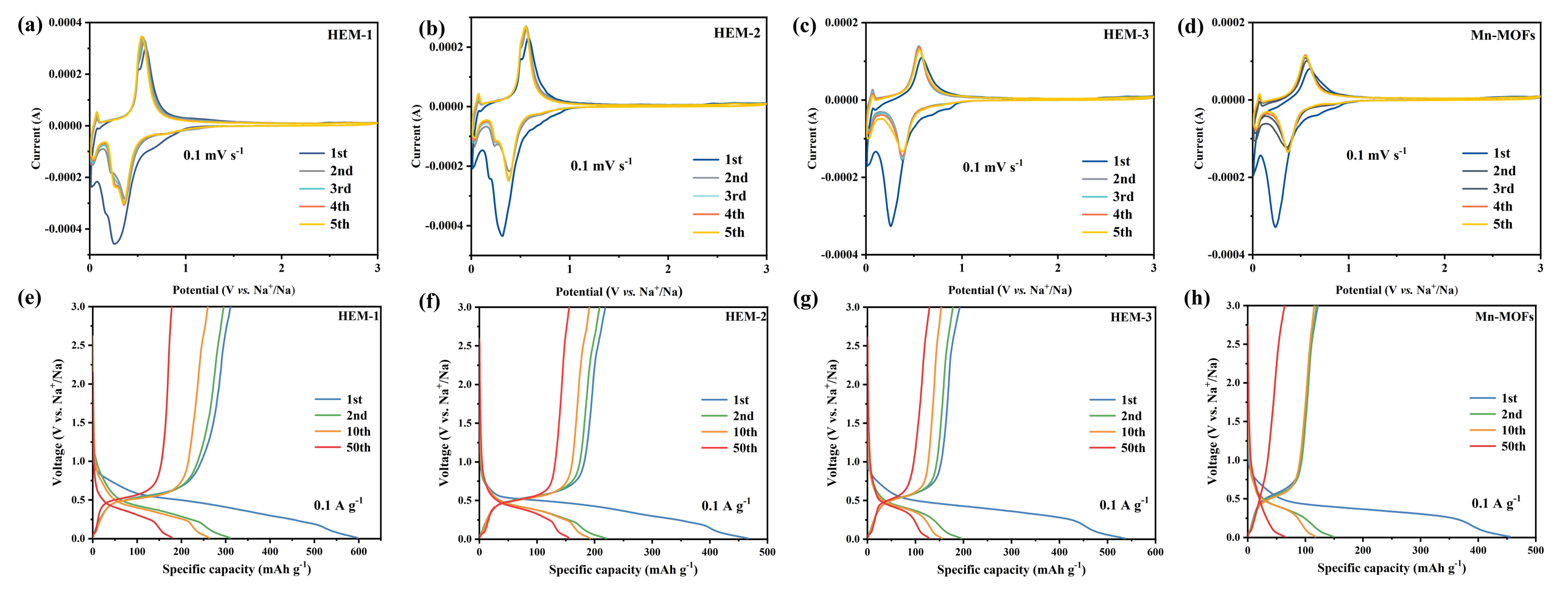


**Fig. S14 a-d.** CV curves at 0.1 mV s^-1^ of MOFs. **e-h.** Discharge-charge profiles of MOFs at 0.1 A g^-1^

**
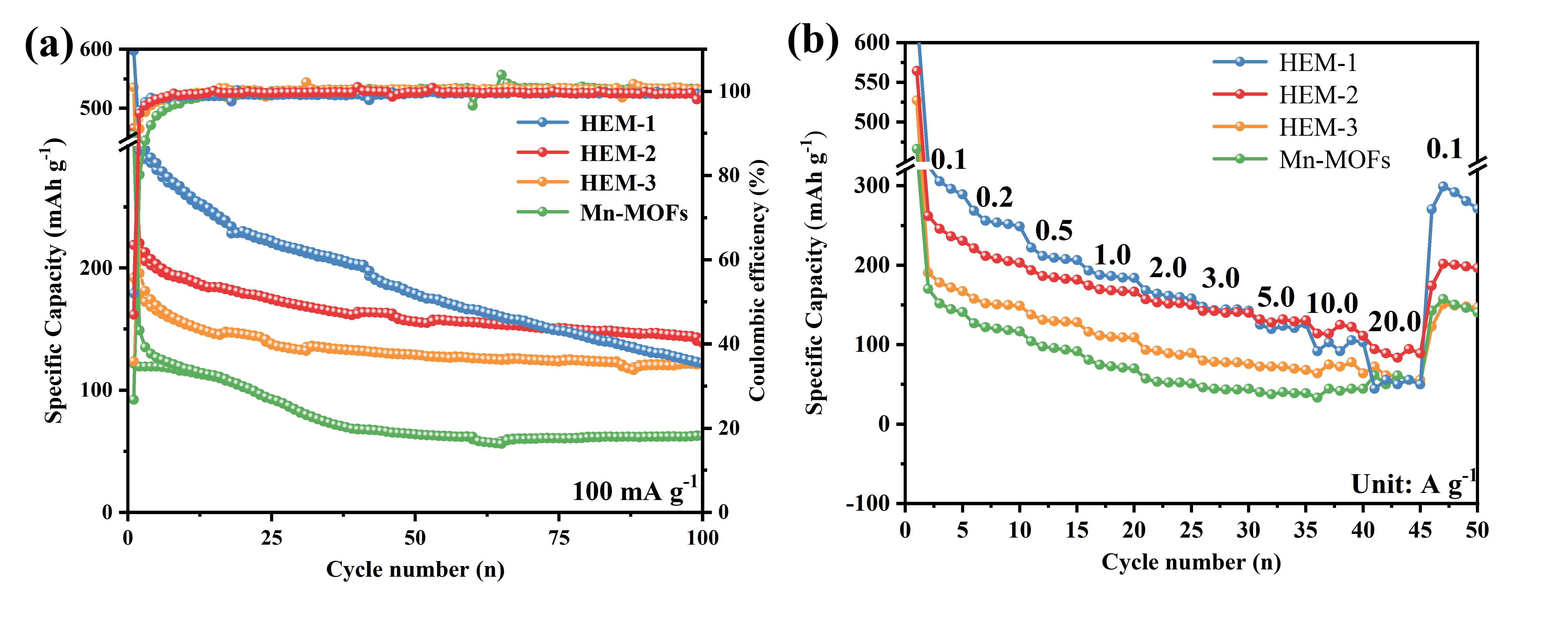
**

**Fig. S15 a.** Cycle performance graph and **b.** Rate performance graph of MOFs materials


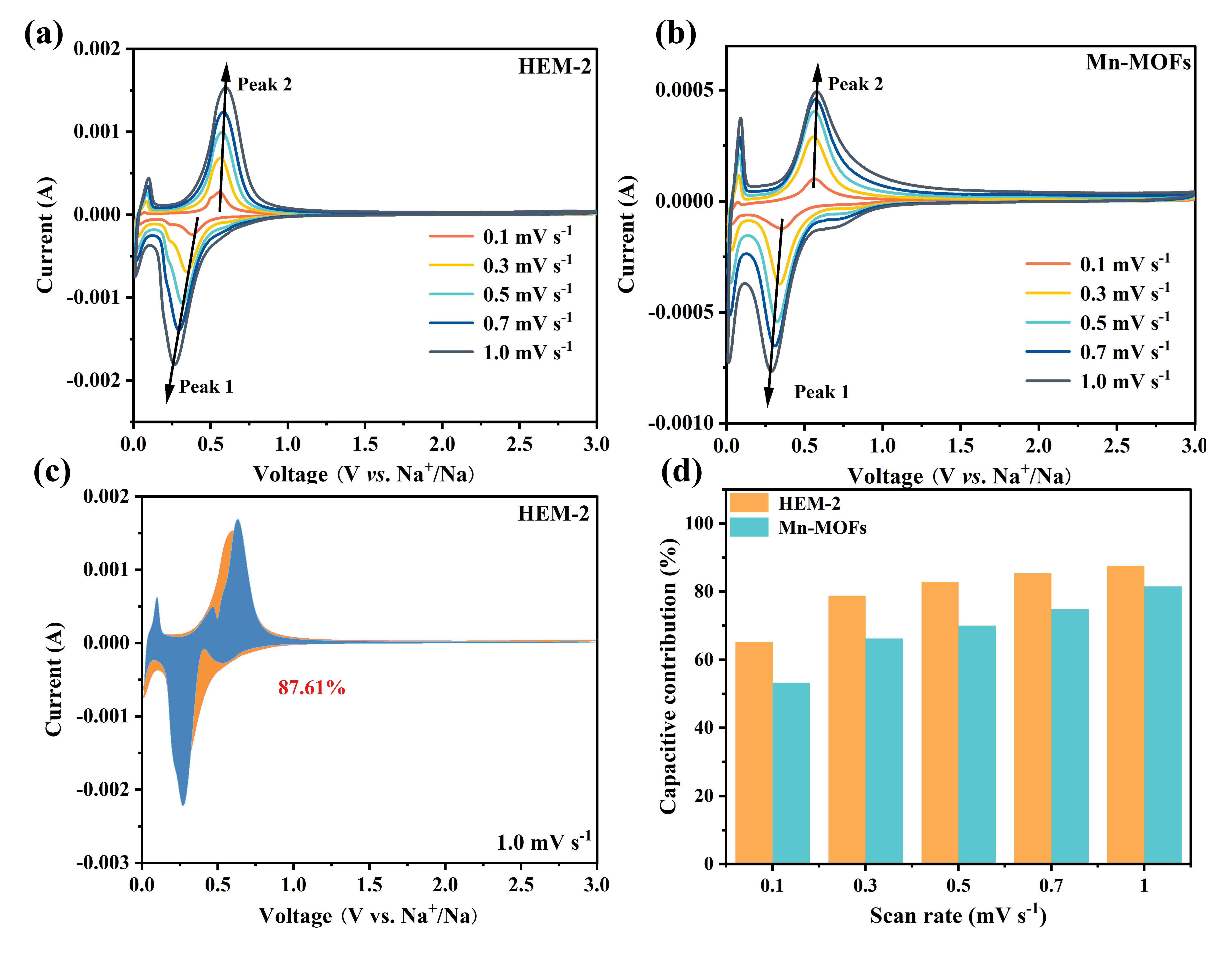


**Fig. S16 Kinetic analysis. a, b.** Cyclic voltammetric curves of HEM-2 and Mn-MOFs at different scanning speeds. **c.** Pseudocapacitive contribution of high-entropy MOFs-2 with a scanning speed of 1.0 mV s^-1^. **d.** Histogram of pseudocapacitive contribution of MOFs materials


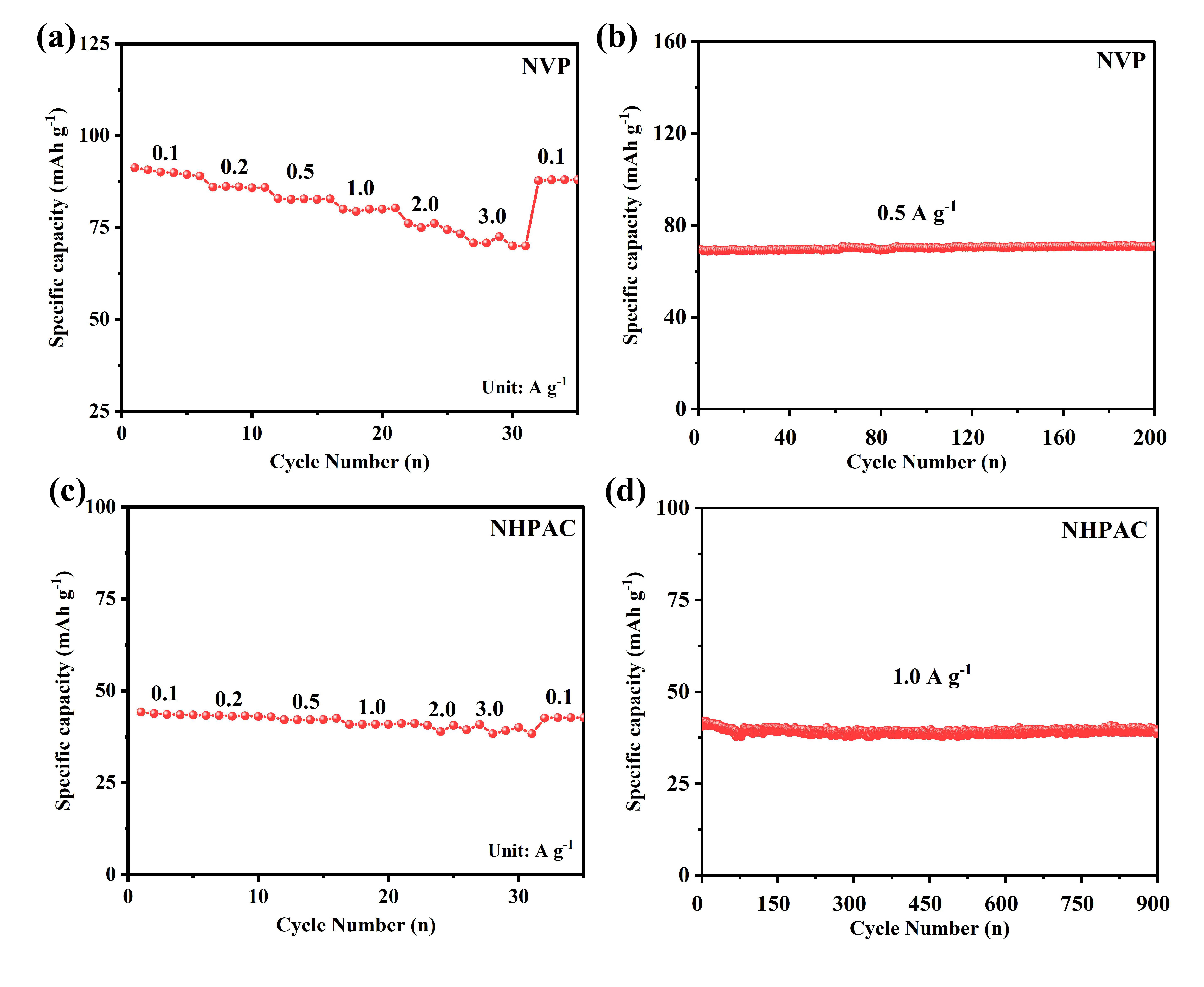


**Fig. S17 a.** Rate performance and **b.** Cycle performance of Na_3_V_2_(PO_4_)_3_. **c.** Rate performance and **d.** Cycle performance of NHPAC


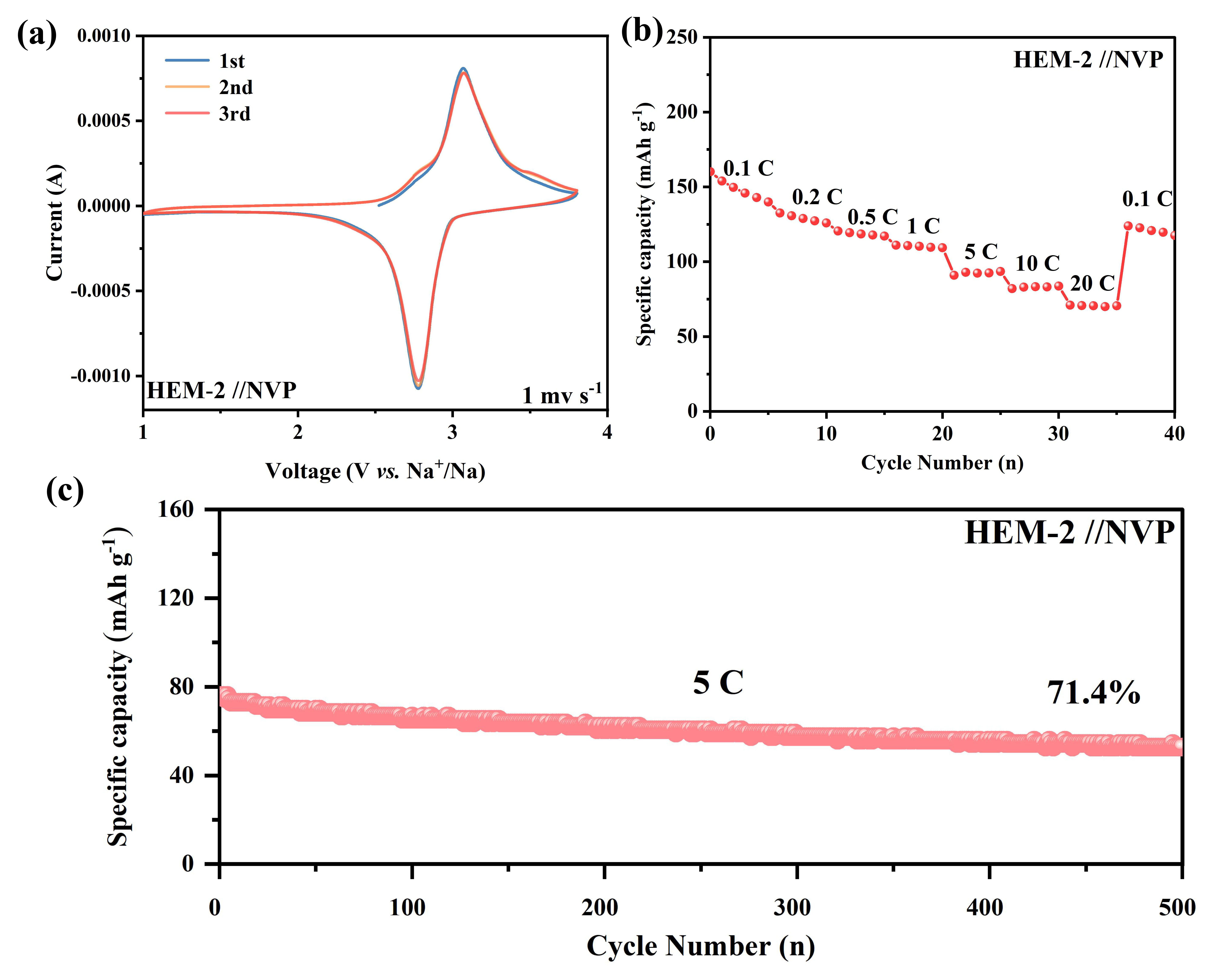


**Fig. S18 a.** CV curve, **b.** Rate performance and **c.** Long cycle at 5C of sodium ion full cell (HEM-2//NVP)


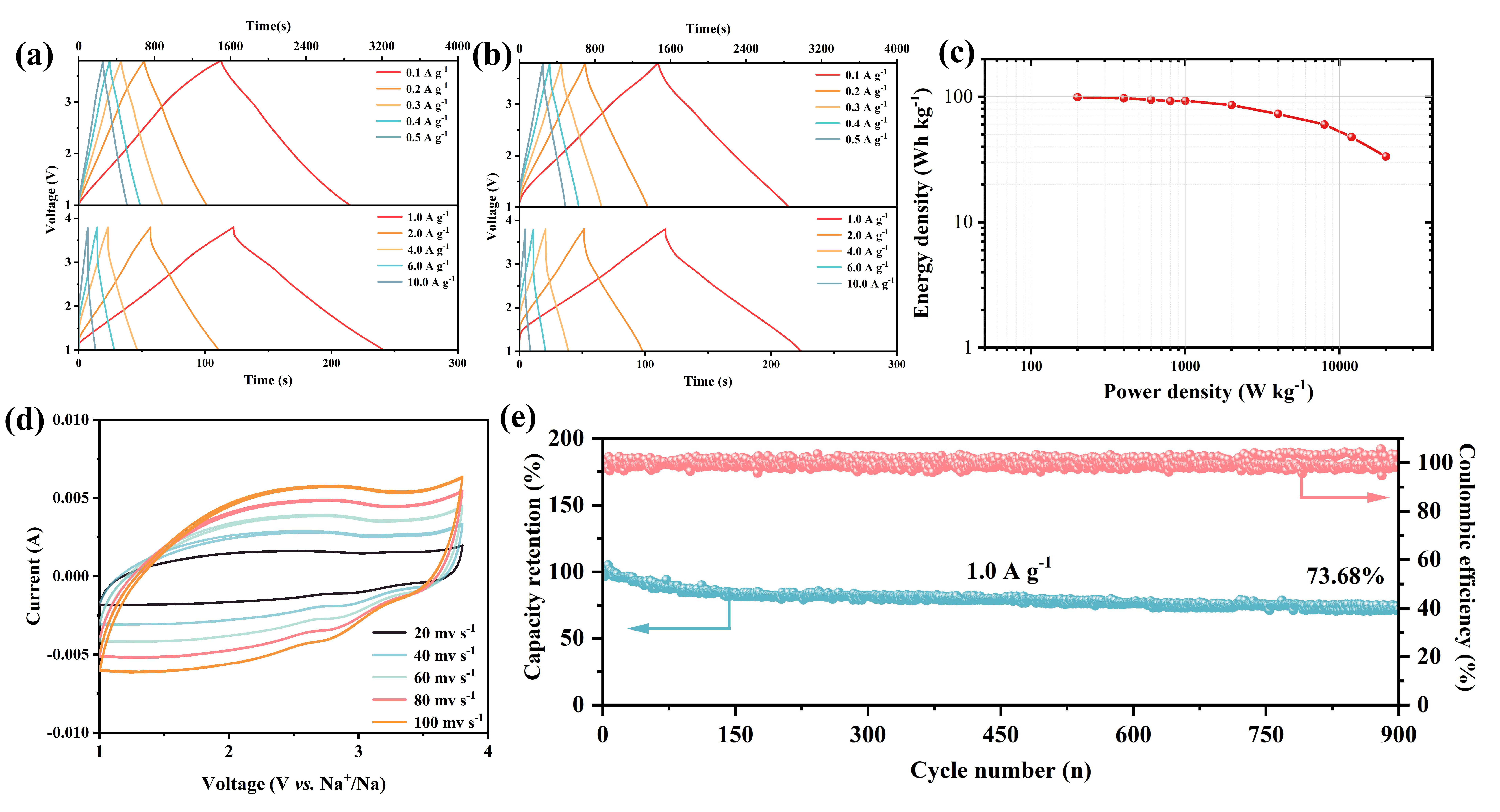


**Fig. S19 a.** The GCD profiles of sodium ion capacitors with the mass ratio of 1:1. **b.** The GCD profiles of sodium ion capacitors with the mass ratio of 1:3. **c.** Energy density and power density data, **d.** CV data and **e.** Long cycle and coulombic efficiency diagrams of sodium ion capacitors (HEM-2//NHPAC with mass ratio of 1:2)


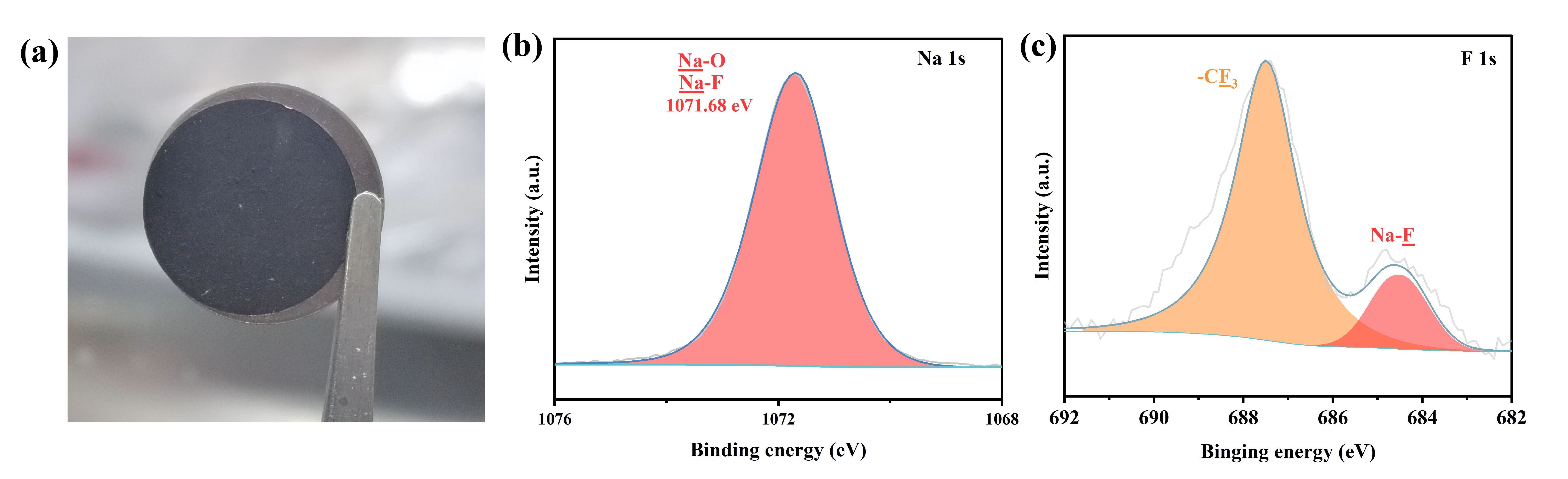


**Fig. S20 a.** The picture, **b.** Na1s, **c.** F1s of HEM-2 electrode of sodium ion capacitor after 900 charge/discharge cycles

**
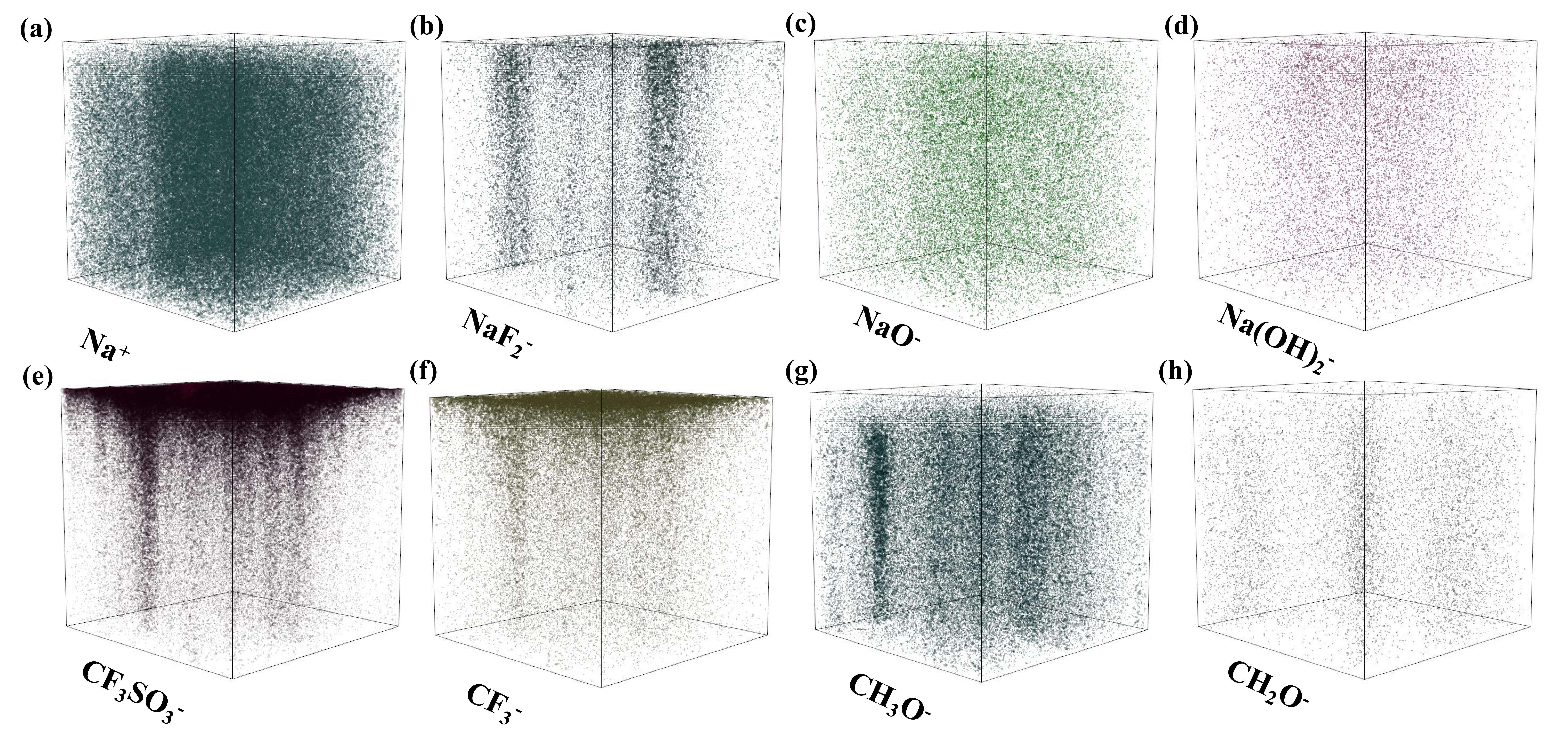
**

**Fig. S21** 3D rendering models of **a.** Na^+^, **b.** NaF^-^, **c.** NaO^-^, **d.** Na(OH)_2_^-^, **e.** CF_3_SO_3_^-^, **f.** CF_3_^-^, **g.** CH_3_O^-^ and **h.** CH_2_O^-^ for Mn-MOFs electrodes

**
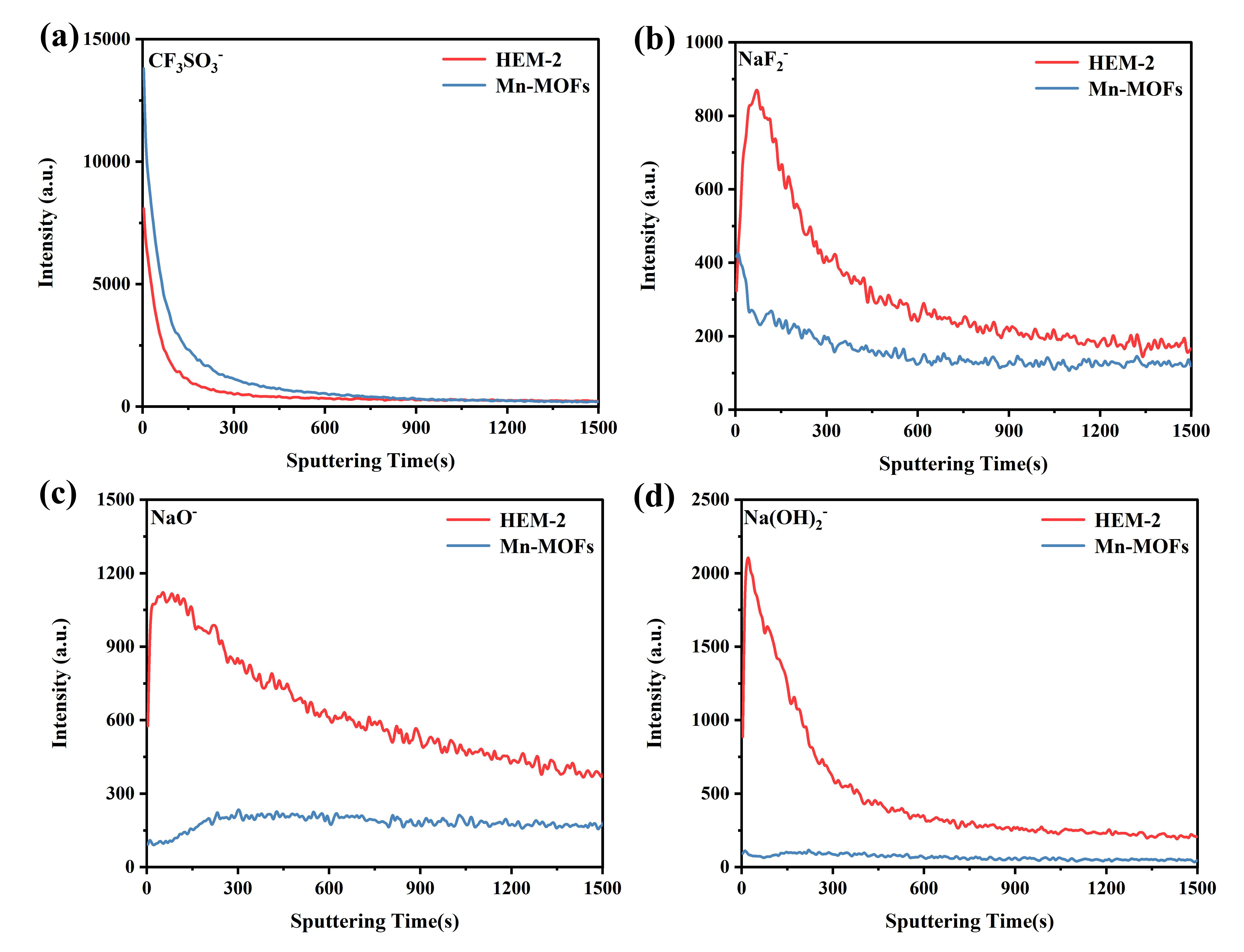
**

**Fig. S22** TOF-SIMS depth profiles of **a.** CF_3_SO_3_^-^, **b.** NaF^-^, **c.** NaO^-^, **d.** Na(OH)_2_^-^ in the HEM-2 electrodes and Mn-MOFs electrodes





**Fig. S23 Elemental influences on electrochemical performance. a-d.** CV curves at 0.1 mV s^-1^. **e-h.** Discharge-charge profiles at 0.1 A g^-1^. **i-l.** Cycle stability of MnCo-MOFs, MnNi-MOFs, MnCu-MOFs and MnZn-MOFs, respectively


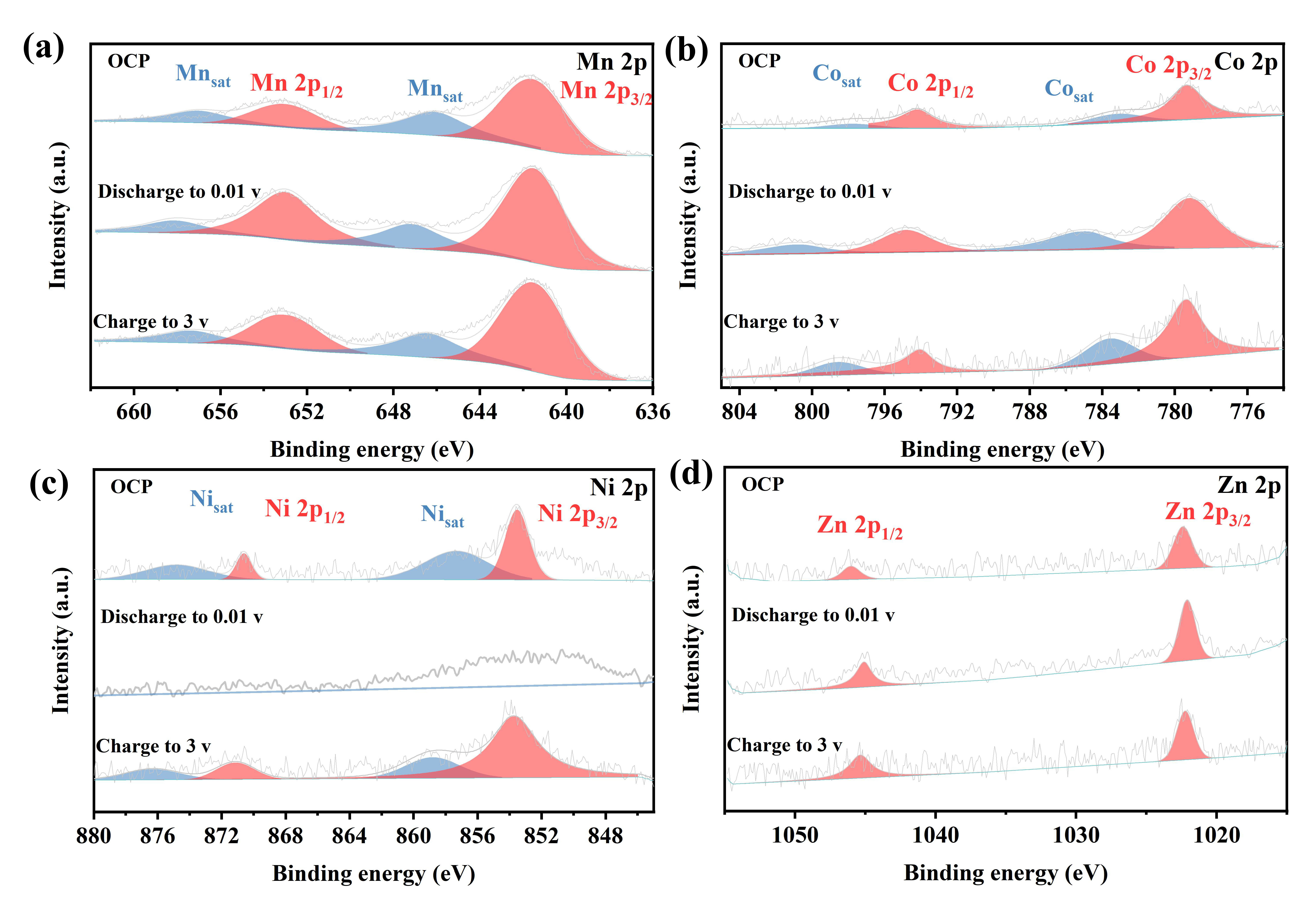


**Fig. S24 a-d.** XPS of Mn_2p_, Co_2p_, Ni_2p_ and Zn_2p_ in different charging and discharging states

**
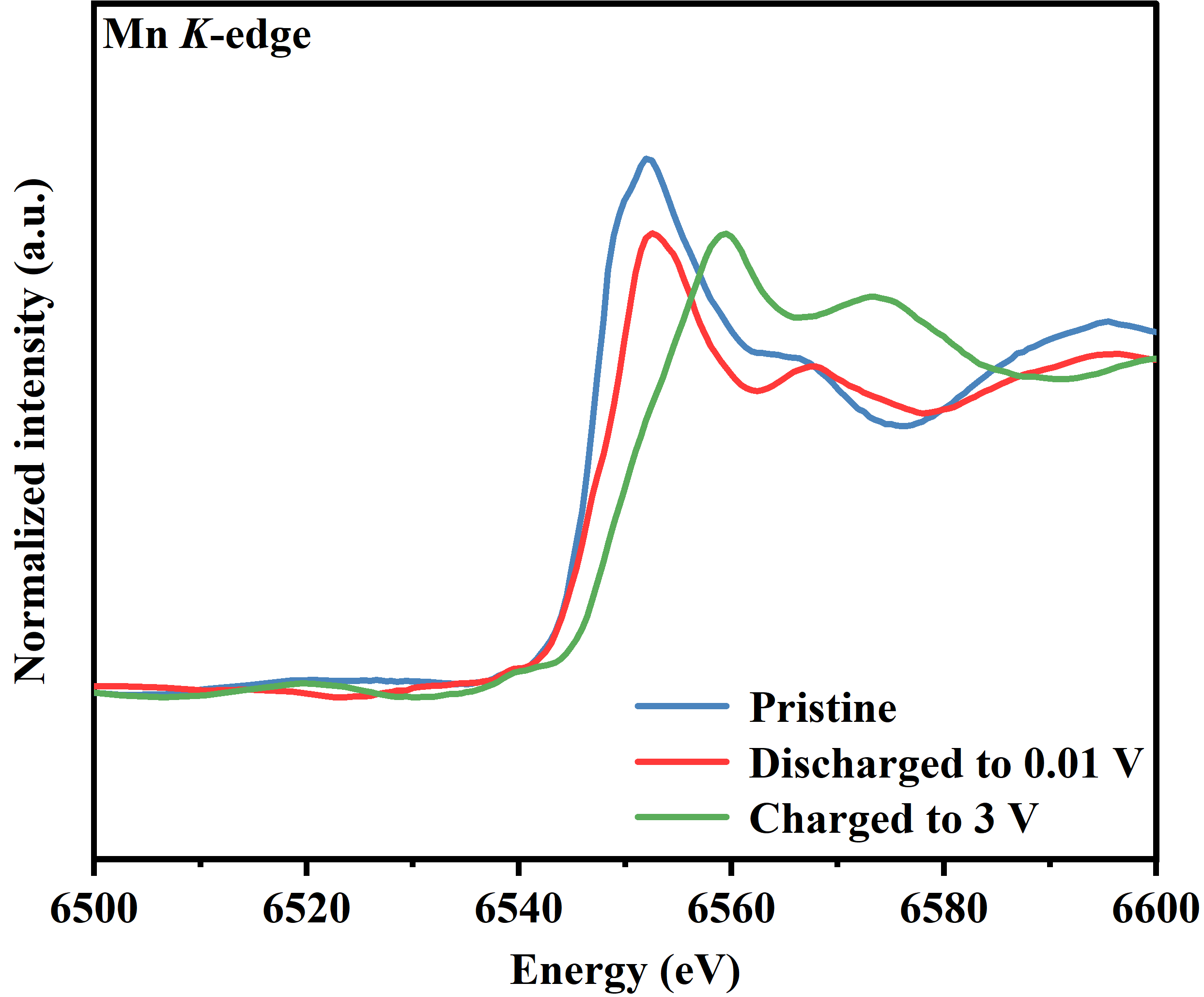
**

**Fig. S25** Mn K-edge of Mn-MOFs in different charging and discharging states

**Table S1** The data of inductively coupled plasma atomic emission spectrometry (ICP-AES)

| Sample | MC of Mn  (mg/L) | MC of Co  (mg/L) | MC of Ni  (mg/L) | MC of Cu  (mg/L) | MC of Zn  (mg/L) | Atomic Ratio (Mn:Co:Ni:Cu:Zn) |
| --- | --- | --- | --- | --- | --- | --- |
| HEMs-1 | 2.848 | 0.899 | 1.299 | 1.326 | 0.610 | 4.3:1.3:1.9:1.7:0.8 |
| HEMs-2 | 68.95 | 8.037 | 12.15 | 17.66 | 9.725 | 6.2:0.7:1.0:1.4:0.7 |
| HEMs-3 | 5.390 | 9.669 | 0.406 | 0.453 | 3.587 | 8.4:0.3:0.6:0.6:0.1 |

MC = Mass Concentration

**Table S2** The data of inductively coupled plasma atomic emission spectrometry (ICP-AES)

| Sample | MC of Mn(mg/L) | MC  of M(mg/L) | Atomic Ratio of Mn to M |
| --- | --- | --- | --- |
| MnCo-MOFs | 65.67 | 34.99 | 6.68:3.32 |
| MnNi-MOFs | 7.53 | 4.13 | 6.61:3.39 |
| MnCu-MOFs | 74.71 | 79.77 | 5.20:4.80 |
| MnZn-MOFs | 60.72 | 22.24 | 7.65:2.35 |

MC = Mass Concentration M = Co/Ni/Cu/Zn
